# Supplementary figures and images for: Continuous monitoring and machine vision reveals that developing gerbils exhibit structured social behaviors prior to the emergence of autonomy
Source: PLoS Biol. 2025 Sep 8;23(9):e3003348. doi: 10.1371/journal.pbio.3003348 (PMC12416696; doi:10.1371/journal.pbio.3003348)

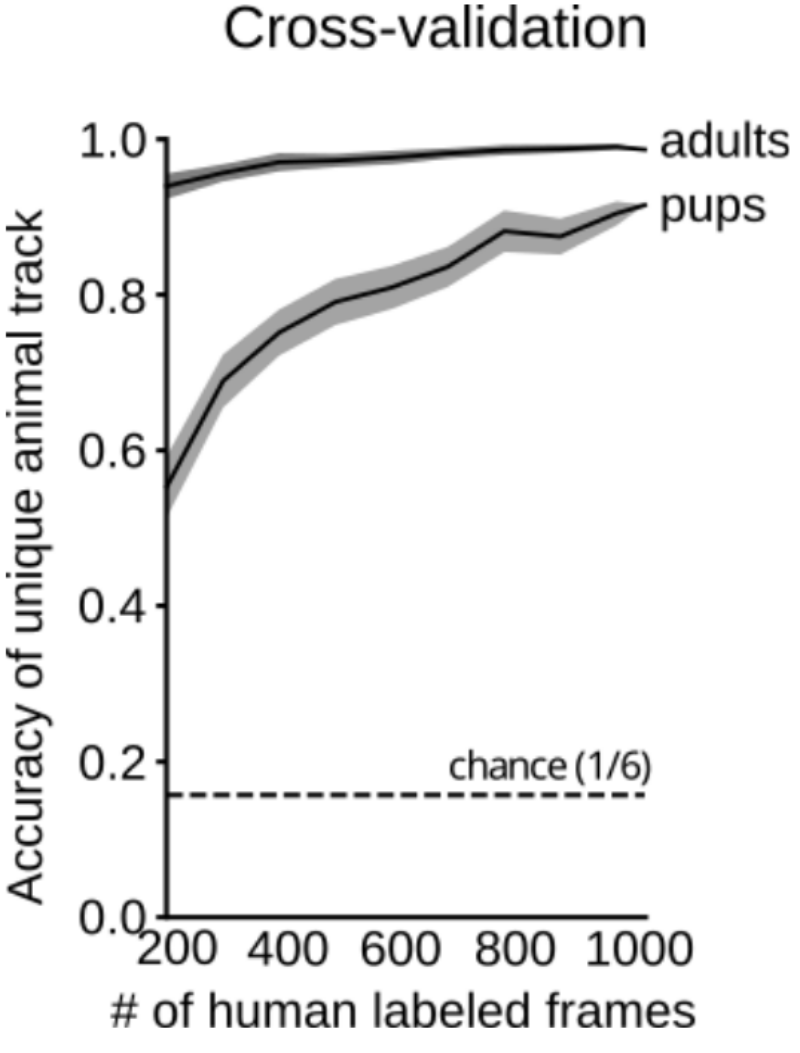

Supplement: S1 Fig — We labeled 1,000 frames per cohort under both day and night conditions (total 6,000 human labeled frames across three cohorts), and studied the accuracy of the animal tracking algorithm (SLEAP) as a function of the number of frames (x-axis) trained. We performed this analysis 10 times per training set size by shuffling the human labeled datasets (except the 1,000 – dataset where we held out only 50 frames and trained on 950). Accuracy of unique animal identification improves asymptotically with the number of human labeled frames in the dataset. (TIF) [file pbio.3003348.s001.tif]

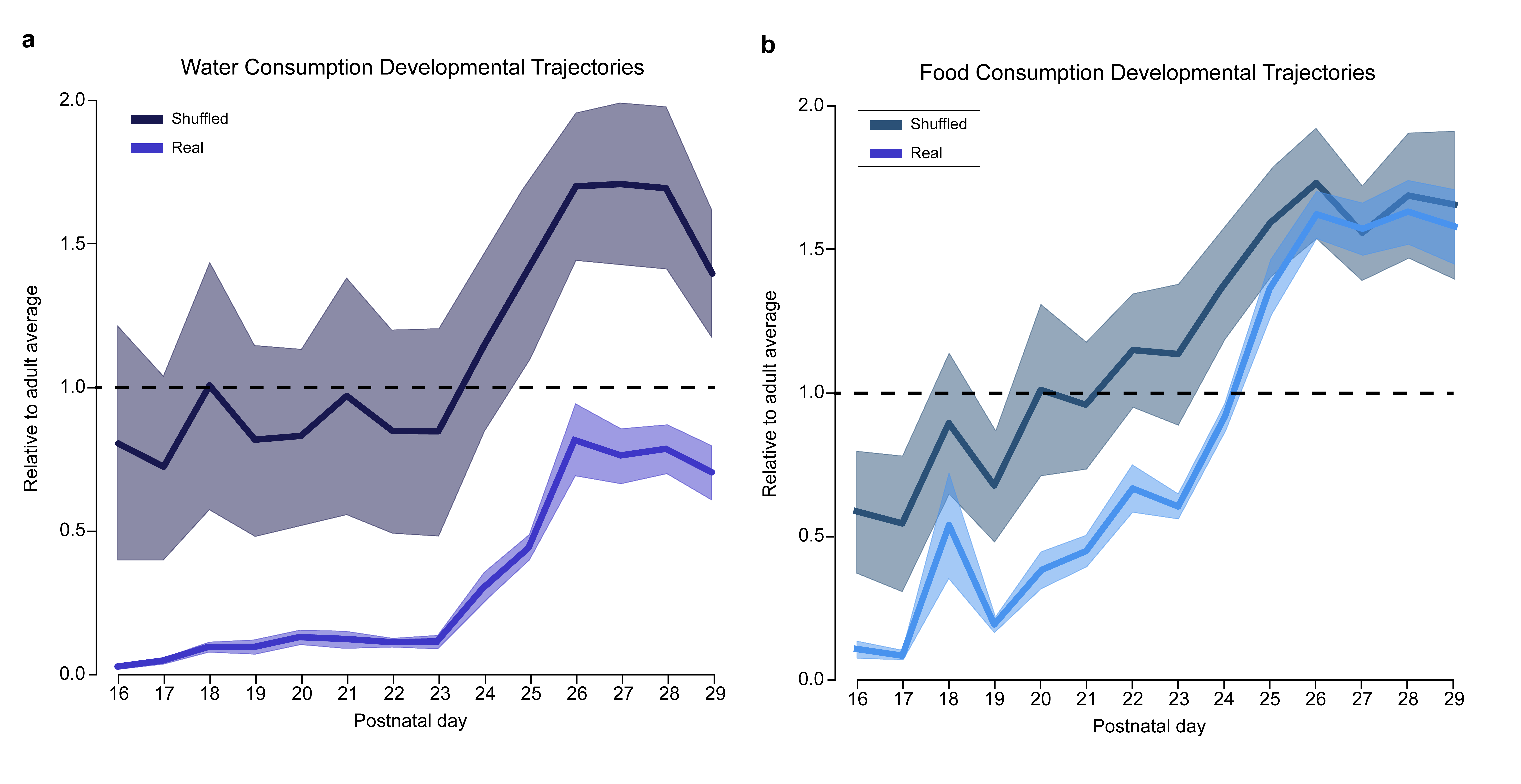

Supplement: S2 Fig — To visualize the uniqueness of the developmental trajectories, we plotted water (a) and food (b) consumption behaviors when pup versus adult identities for correct or “real” identity tracking versus identity shuffled condition. Shuffling identities results in behaviors appearing closer to adults and with higher variance (shading is SEM). (TIF) [file pbio.3003348.s002.tif]

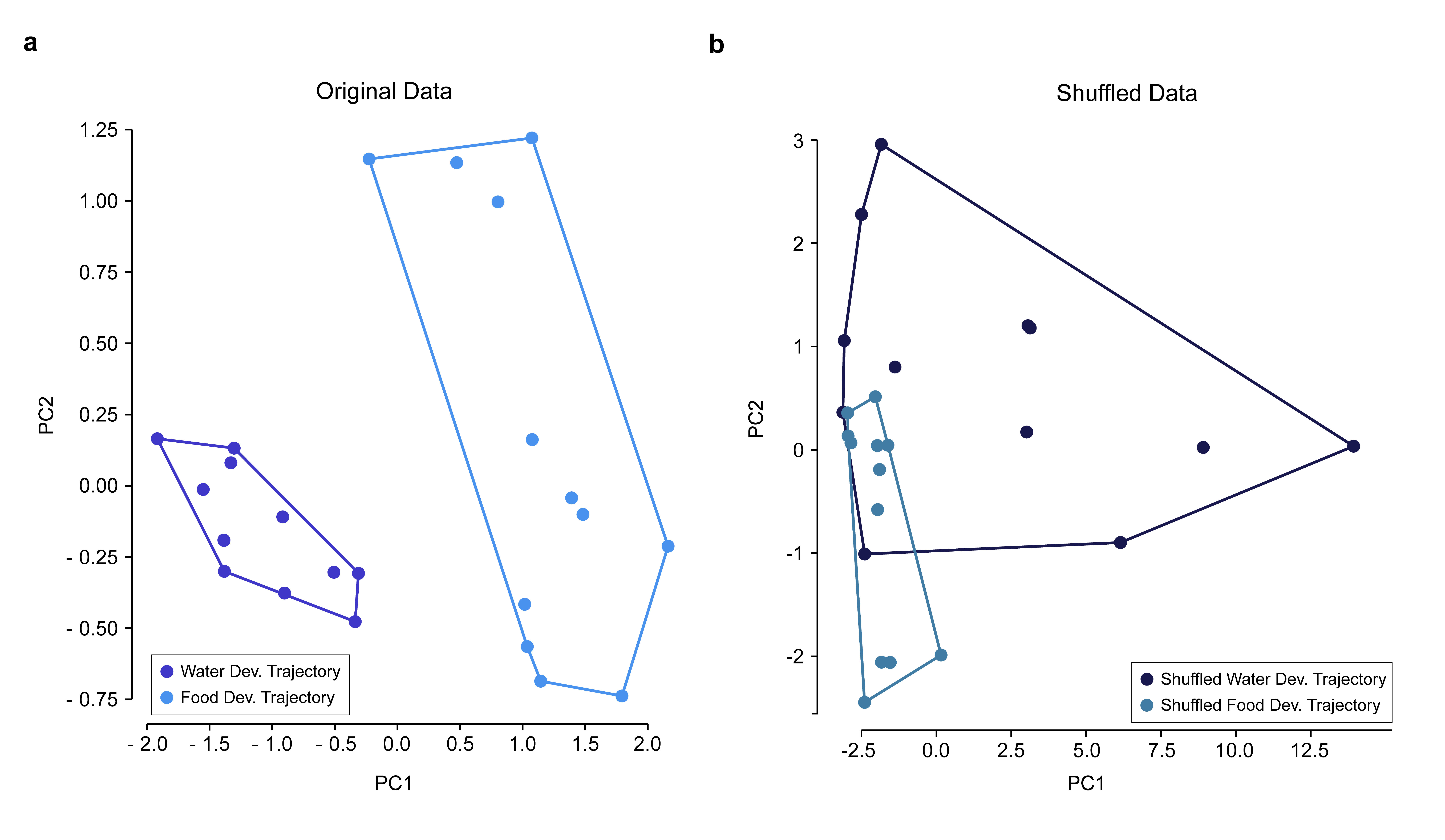

Supplement: S3 Fig — Similarity of intra- versus inter-behavior trajectories over time visualized with principal component analysis (PCA). The individual developmental trajectories (14 Days = 14 Dimensional) for all pups (n = 12) is visualized using the first two PCs of these datasets. (a) When taking into account unique identities developmental trajectories (points) and convex hull (enclosed areas) for water and food proximity in PCA space show significant separation. (b) When the analysis shown in panel (a) is repeated, but with unique animal identity shuffled, substantial overlap is observed. (TIF) [file pbio.3003348.s003.tif]

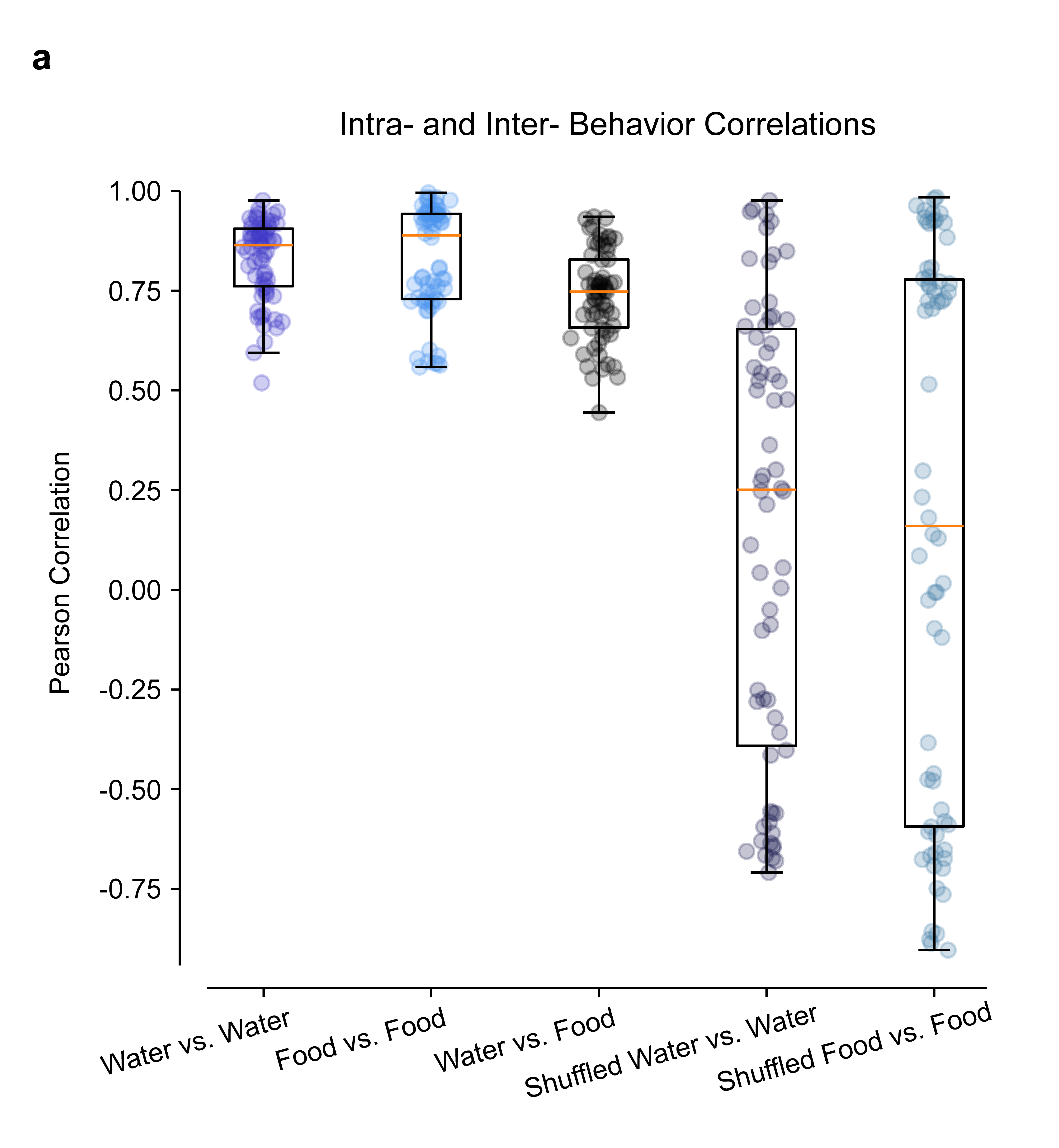

Supplement: S4 Fig — Distributions for all pair-wise individual pup developmental trajectories over 14 days of development are shown for proximity to water and food sources. Within each behavior class, the correlations are much higher for real versus identity-shuffled data. Between behavior correlations, such as water versus food proximity, were lower than within behavior correlations, but significantly higher than those computed for shuffled conditions. We note that correlation between food and water development trajectories was relatively high because both behaviors are generated by the same causal factors, including the increased drive to seek independent sustenance, which unfold on a similar time scale. (TIF) [file pbio.3003348.s004.tif]

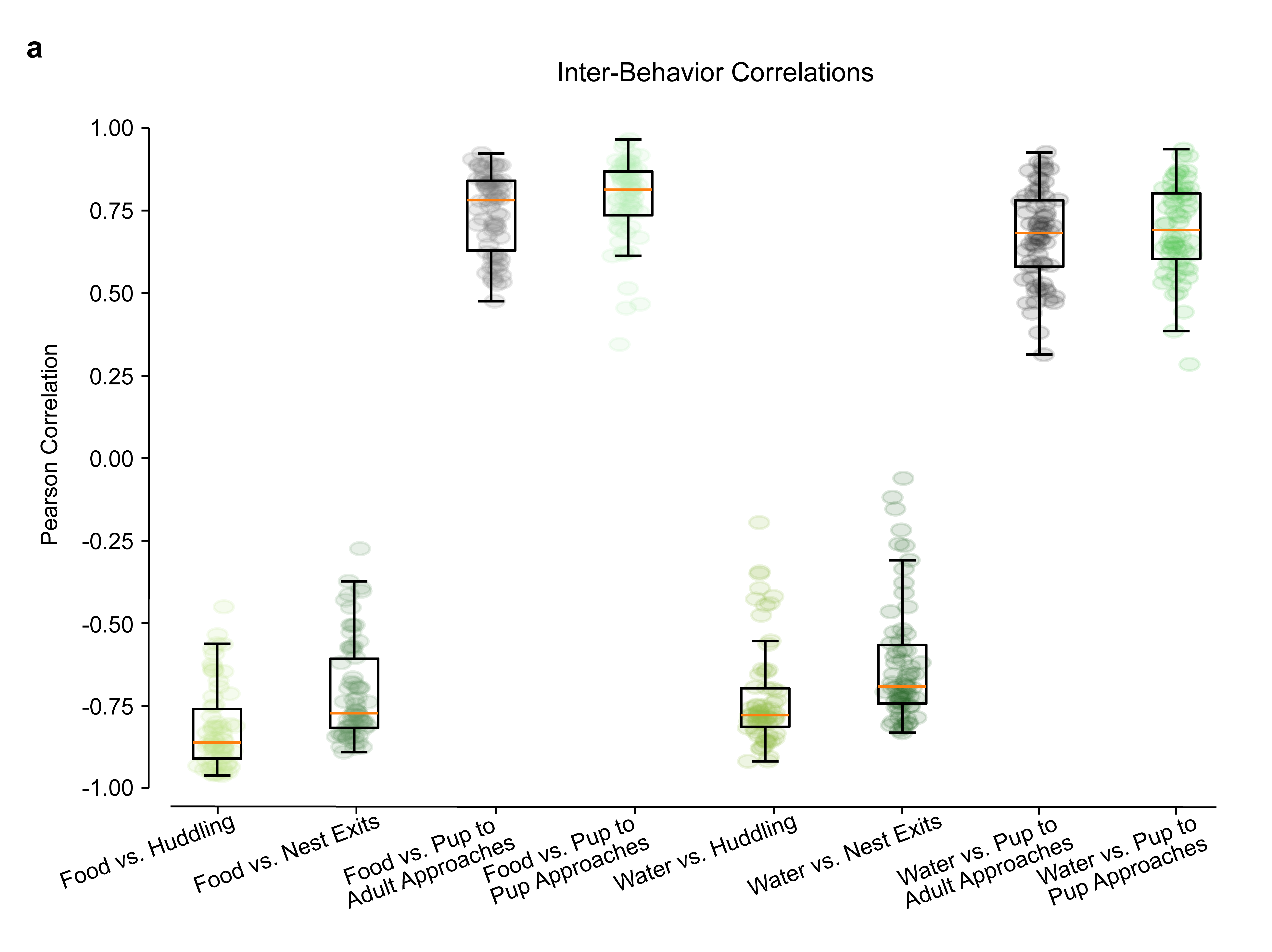

Supplement: S5 Fig — Comparison of food and water proximity to other behaviors yields positive or negative correlations. This result is due to the Pearson correlation between a coarse measure of similarity that relies on the relative value of points in a time series to the mean. As many of the behaviors increased or decreased monotonically in similar ways, we did not view these correlations as a sufficiently sensitive method of analysis in our study. (TIF) [file pbio.3003348.s005.tif]

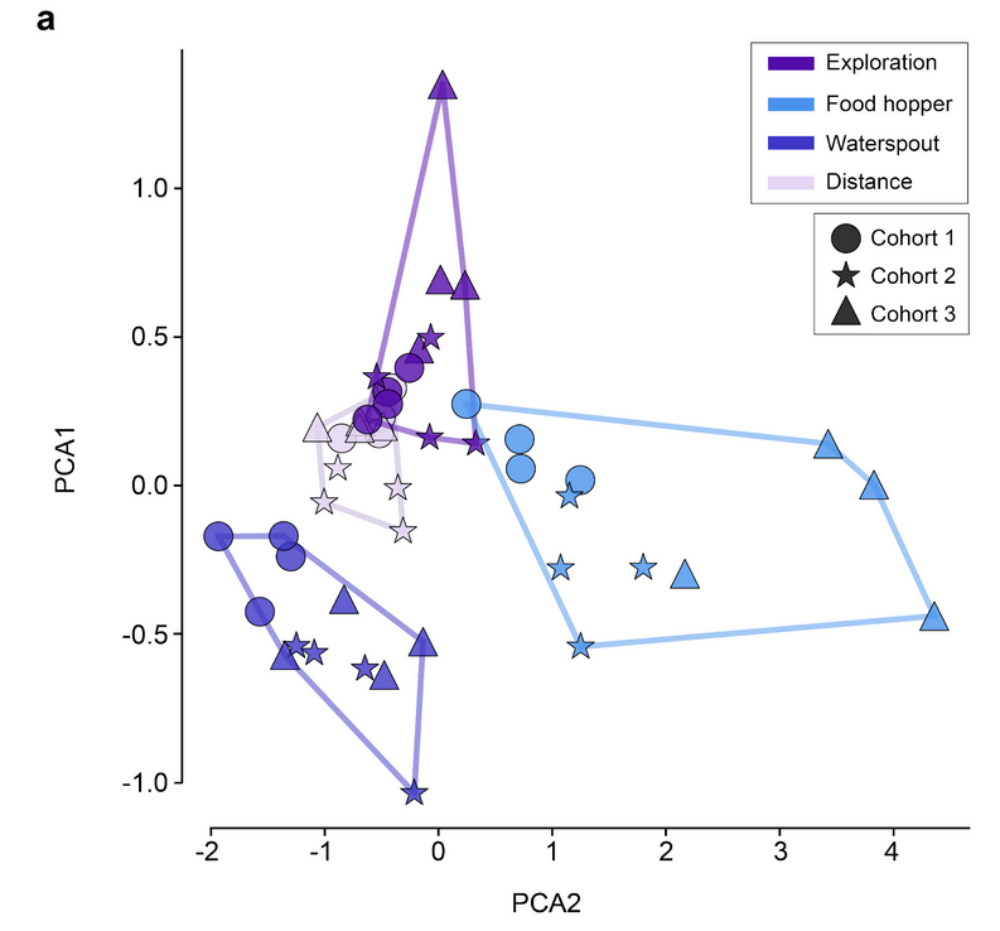

Supplement: S6 Fig — We performed a reanalysis of the data plotted in Fig 2C, but with members of the same cohort displayed as different symbols. There is some grouping of gerbil pup behaviors by cohort number (we used a 2D PCA and a different projection angle to improve visualization). This partial clustering is indicated by the proximity of trajectories (i.e., individual points) for gerbils from similar cohorts (denoted by symbol). However, our primary conclusion is preserved: pups from the same or different cohorts still preserve distinct behavior trajectories (in this higher-dimensional space) when compared across behaviors – and the findings are not drive by single cohort idiosyncrasies. (TIF) [file pbio.3003348.s006.tif]
