# Supplementary material for: Continuous monitoring and machine vision reveals that developing gerbils exhibit structured social behaviors prior to the emergence of autonomy
Source: PLoS Biol. 2025 Sep 8;23(9):e3003348. doi: 10.1371/journal.pbio.3003348 (PMC12416696; doi:10.1371/journal.pbio.3003348)
Supplement: Attachment — Submitted filename: PLOS_response_to_reviewers_PBIOLOGY-D-24-01618.pdf [file pbio.3003348.s007.pdf]

Dear Roli Roberts and reviewers

We thank the editor and the reviewers for their detailed comments. We also thank the journal for identifying sufficient content to our work and agreeing to review our manuscript.

It appears that the decision to reject the paper was based almost solely on the argument that it was underpowered. In our response below we present the rationale and statistical evidence that this criticism is empirically incorrect. We also agree with several criticisms, including that sex-based analysis is under-powered and provide corrections to our work and additional analysis.

Summary of our response

1. Our study is a naturalistic home-cage-monitoring (HCM) study which tracks developing gerbil pups over a period of 14 days from the time point at which they leave the nest until they become independent and would be weaned. The work carried out was interdisciplinary requiring a variety of methods and different expertise including: developmental science and experiment design, development and application of machine-vision tools and analysis using custom algorithms to measure behavior trajectories in high-dimensional space and dimensionality analysis to track social complexity. While we very much appreciate the time and effort that each reviewer clearly put into their constructive comments, we were somewhat surprised by the absence of comment (pro or con) on what we believe to be a unique data set and the complexity of multi-agent tracking across many days. To the best of our knowledge there are no similar works present in the behavior science literature.

2. Regarding our findings on sex-diferences in pups and the lack of sufficient power - we agree with the reviewers that although consistent with other studies, our findings may have weaker support and have removed this analysis and figures from our study. We also want to clarify that the unbalanced pup sex-groups (9 females and 3 males from N=12 pups) resulted from the sex-determination practice at our lab. In particular, the standard practice is to wean and separate the animals by sex at P30 and then resex at P45 - whereas our study ran from P16 to P29. We hope that future studies will ensure a more balanced sex-distribution in the pup litters.

3. Re: the generalizability of our work, we agree with reviewers that the shape and limited size of our home cages may have constrained the natural behavior of gerbils and have made some clarifications in our discussion. We note that home-cage-monitoring studies, however, are increasingly common and our cages were significantly larger than such studies with the floor area being approximately 5 times the floorspace of the standard gerbil family home cage and more than double the height. Nonetheless, we have added text to our Discussion regarding these limitations as follows
    a. However, although the cage area was approximately 5-6 times the size of regular holding cages, it was still significantly smaller and constrained than the natural habitat of gerbils and it is possible that developmental trajectories are different with orders of magnitude larger areas to explore. On this point, we also note that several of the behaviors tracked are correlated due to natural increases in behavior over the period that we studied and decreasing these confounds would require different experimental design, for example, where the natural behavior of developing pups would be constrained to capture possible causal links between the individual behaviors.

4. Regarding the major criticism of our study, that: the number of cohorts (N= 3) in our study was insufficient, we're puzzled by this assessment because the family cohort is not the central independent variable of our study and **does not constitute the sample size upon which the vast majority of our conclusions are based.** Rather, all of our analyses and statistical tests were based on the total number of juveniles (n=12) and total number of adults (n=6), for a total of 18 animals. To our knowledge, there is no hard evidence to support the idea that gerbil (or any rodent) families differ quantitatively from one another across the basic measures that we considered: eating, drinking, distance traveled, proximity, approach, and so forth. We do agree that a research program designed to test such a premise would have to collect data from many families, although one would have to conduct an *a priori* analysis to make that determination. Since our study provides metrics from 6 adults and 12 pups, it could actually serve as a reasonable foundation for such an *a priori* analysis.

5. The validity of our analysis is also supported by power analysis which shows that given the substantial differences (mean and variance) in behavior between adult gerbils and pups in early development, **only 3 individuals per group are required to provide a power of 80% and significance level of 0.05.** We apologize for not including this in our original study, and we have now added it to our Methods. We also discuss this in more detail below.

6. We also point out that 18 animals for longitudinal behavior studies is large in comparison to other developmental publications, most of which harvest a few hours of data from each subject, and few of which even divulge the families to which each animal belonged. For example, a study that is perhaps most comparable to ours tracked groups of 4 adult mice for 4 consecutive nights at 12 hrs/night (Shemesh et al., 2013; https://elifesciences.org/articles/00759). In total, that study tracked four mice yielding approximately 48 hrs of animal tracking. Our study tracks families of 6 gerbils over 300 hours each, for a total of ~1000 hours of animal tracking. As far as we have found, there are no other similar studies to ours that overcome the challenges of multiple-rodent tracking and analysis at this scale. We are in the process of releasing the datasets (video and tracking) to the community as part of our peer-review publication process.

7. To address concerns around whether more animals are required to ensure the accuracy of our tracking and thus generalizability - we had carried out extensive cross-validation tests to evaluate the accuracy of machine-vision tracking vs the number of human labeled frames. We provide this information in our Methods section under Tracking-Features. We have generated the figure below that shows how deep learning machine vision methods capture identity with high accuracy.

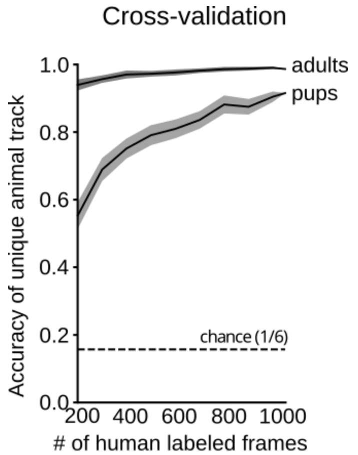

Supplementary Fig 1. 10-fold cross-validation evaluation of accuracy of animal tracking algorithms. We labeled 1000 frames per cohort and day/night condition (total 6000 human labeled frames across three cohorts) and studied the accuracy of the animal tracking algorithm (SLEAP) as a function of the number of frames (x-axis) trained. We did this 10 times per size of training set by shuffling the human labeled datasets (except the 1000- dataset where we held out only 50 frames and trained on 950). Accuracy of unique animal identification improves asymptotically with the number of human labeled frames in the dataset.

8. We did consider the general issue of family differences from a quantitative perspective. Thus, we quantified inter-cohort variances for each behavior and show they were lower than intra-cohort across behaviors. In fact, this was a primary outcome. Therefore, we would turn the question around and ask whether a quantitative argument exists that favors increasing animal numbers as our data did not support such a position.

9. To explain this, we have additionally generated a shuffled vs. real condition to visualize he uniqueness of the developmental trajectories when animal IDs are tracked correctly vs. when identity is lost.

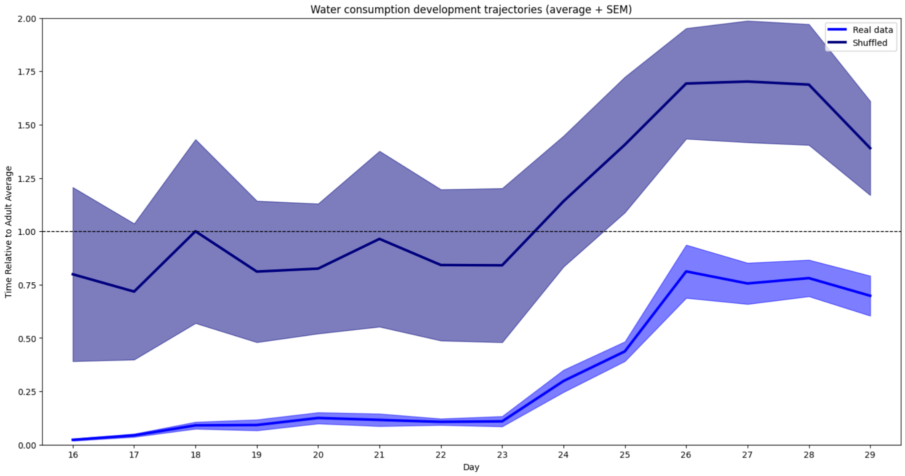

Supplementary Fig #2. Real vs. shuffled development trajectories for water consumption.

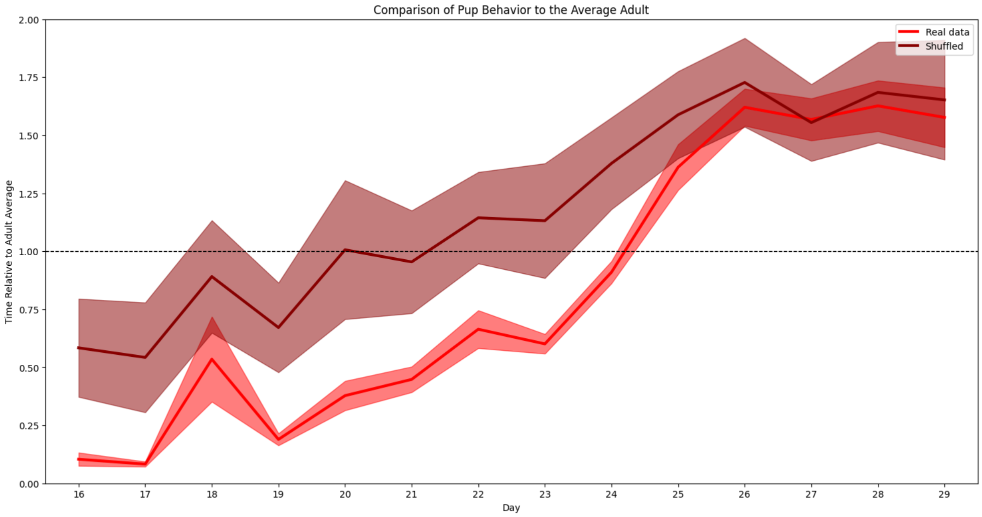

Supplementary Fig #3. Real vs. shuffled development trajectories for food consumption.

10. Both of these figures show that pup behaviours have much closer values (lines) to the adults and much higher variance (shading is SEM).

11. This similarity of intra- vs. inter-behavior trajectories over time can also be visualized further using principal component analysis (PCA). Briefly, the individual developmental trajectories (14 Days = 14 Dimensional) for all pups (N=12) can be visualized using the first two principal components (PCs) of these datasets. These reveal significant separation for real data (where unique identities are preserved) vs. when the identity is shuffled.

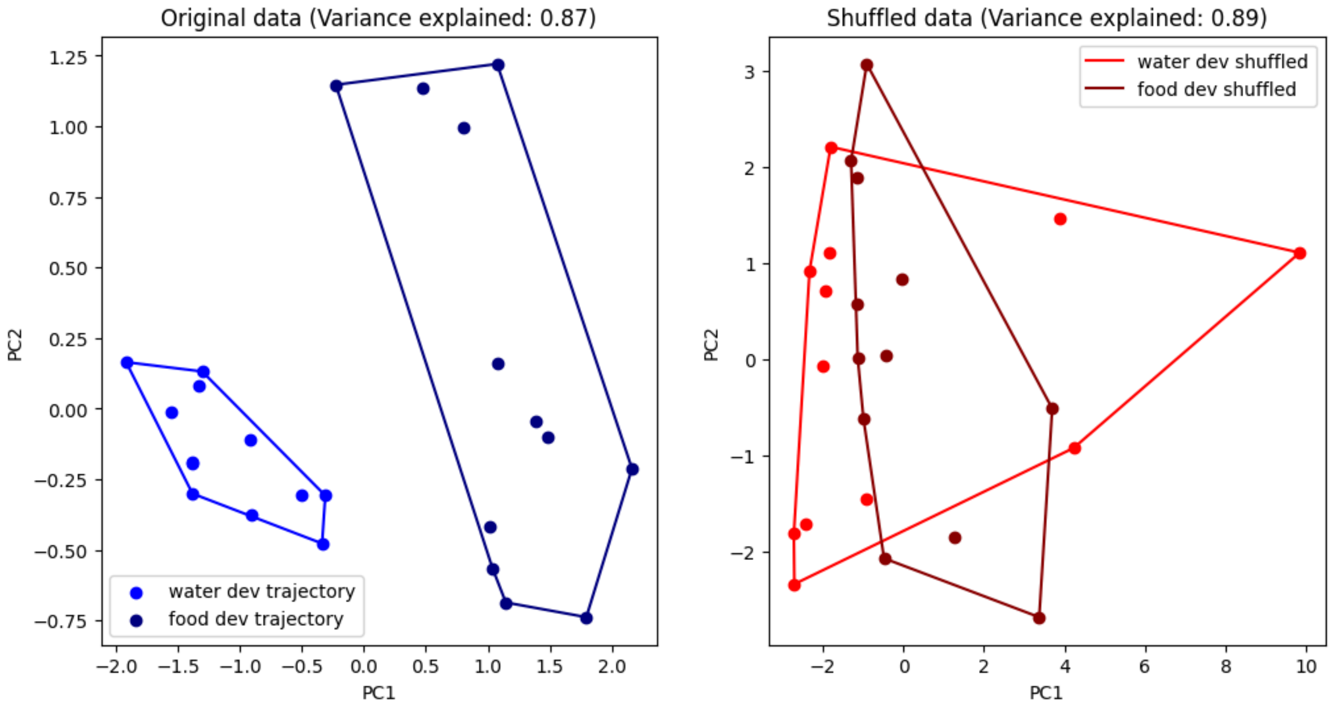

Supplementary Fig #4. Real vs. (identity) shuffled developmental trajectories in PCA space. Left: The developmental trajectories and convex hull for water and food proximity in PCA space. Right: same as left but unique animal identity is shuffled.

12. As reviewer 2 suggested, we can additionally quantify the intra- vs inter-behavior variance by computing the correlations (as one of the reviewers suggested) for real vs. shuffled data. In particular, within each behavior class, the correlations are much higher for real vs. identity-shuffled data.

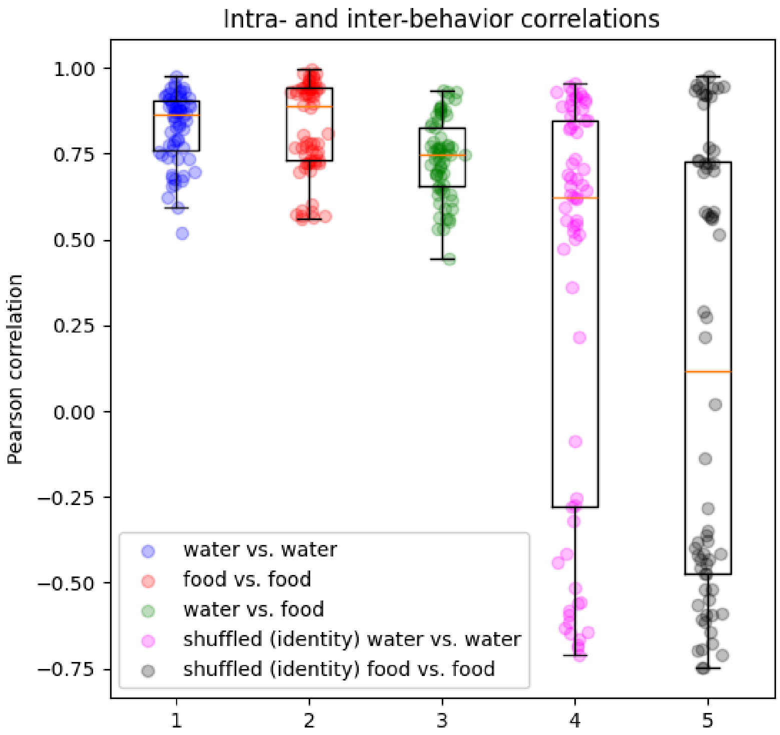

Supplementary Fig #5. Correlation distributions for all pair-wise individual pup developmental trajectories over 14 days of development for water and food.

13. In sum, we view the main and only reason for the rejection of our manuscript as not grounded in the data that we provided, statistical power tests nor the analysis that we carried out to show the uniqueness of animal identity is preserved.

Response to editor

1. You'll see that reviewer #1 likes the approach and the analyses, but thinks that the three replicates are insufficient to draw the conclusions, that in the absence of hypotheses the study is descriptive, and that several confounds mean that causality cannot be established. Reviewer #2 doesn't assess the overall advance, but thinks that any sex-specific findings should be taken "with a grain of salt" because of the low number of replicates; s/he has a long list of queries and concerns. Reviewer #3 is somewhat positive, but thinks that the results should be presented as species-specific, and (like the other reviewers) thinks that causation can't be inferred, and the sex-specific results are very weak (paucity of replicates).

2. We thank the editor for the summary. Our comments are as follows:

    a. Re: three replicates being "insufficient" for the conclusion, we address this in the direct comments above and more below. In sum, the sample size is actually 12 juveniles and 6 adults, and the reviewer did not provide any evidence that the three cohorts from which this sample size was drawn is insufficient.

    b. Re: sex-specific concerns, we agree that these data are preliminary and have decided to remove them from the manuscript, and make this the focus of a future properly powered experiment.

    c. Re: species-specificity of our work, we agree and have re-titled our manuscript to refer to gerbils and made additional edits to our manuscript to clarify that it is possible that gerbil social proclivity may underlie some of our findings.
        i. **Title:** Developing gerbils exhibit structured social behaviors prior to the emergence of autonomy

    d. Re: power-analysis of our study, to achieve a power of at least 80% with a significance value of 0.05 we estimated the required sample sizes for our work are 3 individuals in each group of pups and adults - whereas we had 12 and 6, respectively. Briefly, we estimated these values based on existing literature that shows PDay 16 gerbil pups having little to no movement (they are still in the nest for most of the time) and a small variance of 10% relative to the adult (which was our baseline) and the adult having approximate variance of 20%. Thus for PDay 16 and early development, 3 individuals in each group were sufficient, with 12 and 6 animals in each group providing power of ~90% or better for both 0.05 and 0.01 significance. As we trained our neural networks to identify pups and adults across all days of our study (PDay 16 to PDay 29) our cross-validation analysis applies to all age groups - not just early development - and we found strong evidence that the machine-vision tools generalize sufficiently to keep the same animal identity over the entire window of development in our study. In sum, our data and analysis support that groups of ~3 individuals each would provide sufficient power for this study.

    e. We note that we have also carried out a pilot cohort (cohort #1 for us) which we did not include in this study. This cohort had 2 adults and 2 pups and was used to explore

machine vision algorithms, and supported the earlier publication of multi-animal software SLEAP (Pereira et al 2022; https://www.nature.com/articles/s41592-022-01426-1). This pilot work took over 1 year and enabled the major components of our current study.

f. We also mention here that all video and tracking data will be released on this study as there are other research groups looking for this type of data.

3. During cross-commenting, two of the reviewers noted the shared concerns around the very low number of replicates and correlative/descriptive nature of the study. I discussed these with the Academic Editor, who agreed that, with regret, we will not be able to invite revision of the study, as successfully addressing the concerns raised would entail a significant amount of work (beyond what could be reasonably expected of a revision) and whether the current conclusions would be supported is unclear.

    a. Re: the number of replicates. We note in our comments above that there is no statement that challenges the validity of our statistical analysis.

    b. Re: the study being correlative, or descriptive in nature, we agree, however, PLOS Biology does publish such work. It is obviously not our role to decide this, however, we point out the three cohort study we carried out took nearly 5 years to design and analyze including having to work with machine learning experts (e.g. Pereira et al 2022) to develop the most advanced unique-animal ID tracking software available (i.e. SLEAP). Adding perturbations to our study would have added significant time to our work, likely on the order of years to change protocols, re-run experiments for three or more cohorts. We also point out the positives of a descriptives study which forms the basis of natural, unperturbed behavior, also for future studies.

4. Because producing the required data (including a significant number of further replicates, with a full statistical support for any conclusions) would require substantial cost and effort with an uncertain outcome, we cannot in good faith invite revision of your study. However, given our and the reviewers' interest in the topic, if further work were to allow you to comprehensively address the reviewer concerns outlined above, we would be willing to consider an extensively revised manuscript as a re-submission. The article would receive a new number and submission date and we would evaluate it in view of any related work published in the interim (applying our 6-month scooping protection policy, of course), but we would make every attempt to engage the same Academic Editor and reviewers in its assessment, and the process would not start from scratch but continue from here.

    a. We respectfully question what is meant by “full statistical support for any conclusions.” None of the reviewers pointed out any flaws in our statistical analysis. We provide further detailed submissions above.

    b. In sum, we propose that our modifications including removing the sex-differences results and additional interpretations of our study address the reviewers main concerns.

Response to Reviewer #1:

1. This work focuses on the development and establishment of major classes of behavior in gerbil pups. The study is conducted in semi-natural environments and is based on a continuous analysis of gerbil behavioral patterns. The approach is interesting. It highlights a number of developmental processes during a longitudinal analysis. The analyses are overall well done. A major limitation of the paper is that the experiments were only replicated three times, which clearly limits any discussion of the variability or consistency of the results highlighted. A second limitation of the study is that it is mainly descriptive and that there are very few hypotheses that are explicitly tested.

    a. With respect to three experimental replications, we point out that our findings were statistically verified with respect to numerous metrics. We noted both power analysis and extensive cross-validation that was done to validate our work.

    b. With respect to the descriptive nature of our findings, we partially agree. However, our work was intended to capture the natural development and stereotypy of gerbil pup behavior - and we did not seek to perturb this pathway in the current study. Further studies could be used to explore how perturbation in the ability to socialize in early development affects downstream behaviors - and are beyond the scope of our work.

2. 1: The cage is a key level of analysis, and with only three experimental replicates, the study is severely limited. First, there is no statistic comparing gross quantification (circadian behavior, activity...) at the level of the cage. This is important to understand if the cage level contributes to inter-individual variability. Furthermore, as a consequence of this low number of experiments, at the individual level, behavioral observations were also made on a limited sample comprising 3 adult males, 3 adult females, 3 male pups, and 9 female pups. In particular, one of the cages lacked male pups, significantly limiting the analysis of sex-specific behavioral dynamics: for instance, interactions between male pups were observed in only one cage, underscoring the challenge of drawing comprehensive conclusions about such interactions under these conditions.

    c. Re: inter-individual variability, we have addressed this above and in our comments to the editor. Briefly, we find that intra-behavior variance is much smaller than inter-behavior even when pooling data from all three cohorts. This suggests that behavior development is more likely driven by general processes than individual cohort idiosyncrasies. Given the stability of our tracking across 18 gerbils and findings, we ended our data collection.

3. 2. One of the goals of this work is to study the transition from social to solitary behavior during development. A question that arises is whether these two behaviors are independent in this environment. Due to the restricted environment, the animal has limited options. If an animal decreases its social behavior, it will increase its solitary behavior and vice versa. Therefore, it is difficult to determine causal relationships between events, such as whether foraging behavior results from or is related to early social interactions. This question is inherent in these approaches and does not disqualify the study, but it does require specific analyses to show that the interdependence between measurements is limited (i.e., we're not measuring the same thing

20 times) and that the conclusions are causally correct (perhaps through statistical modeling?). At the very least, it warrants a thorough discussion.

a. Re: the interaction of social and solitary findings - we agree that some measurements, such as time spent exploring outside the nest, correlate with others such as amount of time spent in the food-hopper region. However, our tests show that correlation alone is far from explaining some of our findings. For example, we have evaluated the percentage of time the animals spent in non-salient areas of the home-cages (e.g. a randomly drawn Region-of-Interest; ROI) is significantly lower than in food-hopper and water arenas.

b. We have added to our Discussion that some of our measurements are correlated and that decreasing such confounds would require different experimental design where the natural behavior of animals must be constrained.

4. Minor: - In the first part of the article, it is unclear whether the rapid increase in visits, water or feed dispensers is solely due to exploration. The animals are weaned at P30, but at the same time the authors consider that exploration of the hopper is also a means of learning about nutritional value. Do puppies begin to eat before weaning? Could the increase in food hopper visits also be due to an increase in the animal's needs? The same question applies to water. If there is a rapid reduction in the time spent nursing before P30, then having an idea of the needs (what they eat and what they drink) could be important to understand the emergence of solitary activity.

   a. Re: the increase in food and water area proximity, we did not find similar increases in randomly selected regions-of-interest (ROIs) tested (data not shown). Thus the natural increase in distance traveled and exploration time alone could not account for these results. We also note that pups spent significantly more time in the food hopper area (approximately 2 x as their parents) and we did not have evidence (or found literature) to suggest that they consume 2 x the food of their parents, despite being 25-50% the size of the parents at that age. With respect to water consumption and nursing, we noted that the female adults (i.e. the mothers) decreased their feeding and water consumption at a similar time (1-2 day period) when pups began to consume water significantly more.

5. "Distinct inter-behavior developmental trajectories of territory exploration in gerbil pups." I'm wondering if estimating intra- and inter-individual variance might be an easier way to show that there are individual developmental trajectories. Is this what has actually been done? It is also a bit unclear what the differences between the developmental trajectories are - do all pups follow the same time course with only differences in the starting point of the increase? Finally, in the conclusion it is say that "Across both autonomous and social behaviors we observed stereotyped trajectories in development showing that inter-animal behavior variance is much smaller than inner-behavior variance." This suggests that the observed behaviors are quite consistent across animals, which contradicts the idea of distinct developmental trajectories. This should be clarified.

   a. Re: intra- and inter-behavior variances, we sought to quantify this using high-dimensional overlap analysis (Figs 2C; Methods). Briefly, we showed that the

behavioral developmental trajectories for a specific behavior (e.g. the amount of time spent outside the nest / day) were more similar across pups when compared to another behavior (e.g. amount of time spent in the food hopper region). Based on this analysis, we can conclude that pups do follow similar "time courses" for each behavior than they do across behaviors.

b. Re: the "observed behaviors are quite consistent across animals" contradicting the "idea of distinct developmental trajectories", we hope the above statement helps clarify. We have added the following to our text:

    i. The time courses tracking the development of single behaviors for pups from different cohorts are more similar when compared with the time courses from the same pup but for different behaviors. This supports that the development of individual behaviors have distinct trajectories - rather than being explained by coming from pups in the same cohort.

6. "Similarly, pups were followed out of the nest by parents significantly more than adults on P16. The sentence is not very clear. Are the authors comparing the number of times pups were followed out of the nest by adult to the number of times an adult was followed out of the nest by another adult? Also, what are the percentages relative to? Did the authors calculate the percentage by taking the number of times a pup was followed out of the nest by an adult and dividing it by the total number of times pups left the nest? Or did they divide the number of times a pup was followed out of the nest by an adult by the total number of times any gerbil (adult or pup) followed another gerbil out of the nest?

    a. We thank the reviewer for raising this issue, we agree it was not clear. The statement was meant to indicate how much more likely it was for adult gerbils to follow each other when leaving the nest vs. pups when they left the nest. The goal was to evaluate how much adults care about pup dispersal and potential engage in retrieval (which we did not track) in early development.

    b. We have clarified this statement as follows in the original text "Similarly, pups were followed out of the nest by parents significantly more than parents followed other parents on P16…"

    c. We calculated these values similar to all other values in our study - by normalizing the values relative to the number of adult-adult behaviors. Thus, in this case, the numerator contained the # of times pups were followed out of the nest by adults, and the denominator the # of times adults were followed out of the nest by other adults.

7. The methods used for the social dynamic study are not clearly described. Here are several questions that may reflect my misunderstandings of the methods:

8. I assume that Nvideoframes represents the number of changes in occupancy. Is that correct?

    a. Nvideoframes represents the number of time points in our video recordings, approximately 86400 time points per day of video (in second time bins).

9. The social dynamic study is based on a space-partitioned, 20-dimensional state space. Each of the 20-D vectors contains values between 0 and 6 if six animals are considered, resulting in N possible states (calculated using permutations and combinations). The authors then study the transition matrix between these N states. Could you explain what exactly Figure 5D represents? Does it show the transition from one state to the next?

    a. We did not expect that the gerbil families would explore all possible configurations or arrangements in their environment as this is very large. So we sought to use graph and network analysis to quantify how social behaviors may be more interpretable.

    b. Figure 5D is a graph or network representation of the social states - or configuration - in this 20D state space. Each point in the graph represents a unique configuration (e.g. a vector from 5c) and the edges connecting the points indicate that these unique configurations occurred in sequence. Thus, the graphs can identify which social configurations are present (Fig 5b) and the edges reveal how these configurations change over time. This helps to identify unique properties of such social configurations.

10. Clarify that Figures 5E, F, and G represent the number of states (size) and the exploration network in the case of a single animal (where there are 20 possible states), and similarly for Figures 5G and I. The state space is entirely different when considering N=6 animals and in this case the figure 5E is not correct? I'm afraid that with this presentation people thing that network size is the total number of cells that have been visited.

    a. The reviewer is correct, that Figures 5(e)(f) and (g) are sketches for a single animal. As described in the Figure legend, these are sketches intended to provide sense of what they each measure to readers that are not used to this type of analysis. The reviewer is correct that Fig 5E for 6 animals would look different.

    b. For a single animal, the size of the network is indeed the number of unique locations that a single animal visits. For two animals it is the unique number of two-animal combinations explored. Thus, a single animal could have a maximum network of size 20, while paris of animals could have up to 20*20 = 400 configurations. As shown in Figure 5f, both adults and pup pairs only explore about half this social space.

11. If I understand correctly, the size indicates the number of states visited. If N is the total number of possible states (different for single animals (N=20), adults, etc.), should the size data in Figure 5F be divided by N to allow for a comparison of the different curves? Same for h and j: is it possible to normalize ?

    a. We thank the reviewer for this comment. We had considered normalizing, however, it was not clear whether this helped for interpreting these results. As the number of possible states increases exponentially with the number of animals normalizing would capture this exponential increase more than the absolute metrics we were measuring. This also generates very small values (i.e. y-axis values). We are open to providing the normalized versions of these plots in the final version of the manuscript.

12. For Figure 5H, within a specific group (single, adult, etc.), how are cycles defined? Is it the total number of distinct cycles or the total number of cycles (even if it is the same cycle repeated 6000 times)?

    a. We thank the reviewer for this comment also. It’s the total number of distinct or unique cycles - not the total number of times unique cycles were visited by each social group. We did not measure the latter, but it is perhaps something to add as well. We have a statement clarifying this to our manuscript.

13. These clarifications will help in understanding the methodology and results presented in the study.

Response to Reviewer #2:

1. Summary Main Results. The authors used video to capture behavior in gerbil adults and pups from P15 to P29 in an enlarged home cage using SLEAP for animal tracking and SimBA for behavior classification. In pups, distance traveled and open exploration time rapidly developed between P17 to P25, eventually stabilizing to adult levels. Other behaviors, such as food hopper & water spout exploration rose more slowly early in development, rapidly developed around P22 to P26, stabilizing around P27 to P29. Food hopper exploration exceeded adult levels from P27 to P29, while water spout exploration stabilized slightly below adult levels. Pups tended to spend more time with other pups than with adults and would socialize in groups of 3, with groups composed of pups rather than adults. Social behaviors had a stronger presence earlier in development (~P16 to P18) compared to food & water acquisition behaviors. The authors report sex differences in both adults and pups in behavior. Specifically they report:

2. Adult females spent more time outside the nest and both female adults & pups traveled greater distances than male adults & pups, respectively. Female adults spent more time in food hopper and waterspout regions compared to adult males, while female pups only spent more time in food hopper region compared to male pups. Pups of the same sex spent more time with each other than with the opposite sex. Both adult males and females spent more time with female pups than with male pups. Male pups had shorter episodes of social interaction than female pups. Pups approached adult females more than adult females approached the pups, and pups would more often than not approach the adult female compared to the adult male. I take these results in particular with a grain of salt. The total dataset came from only three litters. There were only 3 males and they were not in all litters so these results are perhaps not reliable. It is also not made clear how sex specific approach behaviors were different from chance (if approaching any littermate) rates as these chance rates were not included in results.

    a. We agree with the reviewer and editor and have removed this part of our study from our updated manuscript. While we sought to maintain the ethology of our work and not disturb or otherwise cull the pups of the adult pairs, we could have done a better job ensuring there are at least one pup from each sex in each cohort. We also point out that the sex of the pups is not generally determined during the early development at our lab. In particular, the standard practice in the lab is to wean and separate the animals by sex at P30 and then resex at P45.

3. To further, measure the complexity of social states, the authors divided the cage floor into a 5 by 4 grid and defined a social state at each recording time point as the number of animals at each location. Complexity of social behavior increased across development for larger groups of animals. Novel social-state configurations were generated from P16 to P24, driven by pup development. Single animals had a greater tendency to exploit existing social configurations, while larger groups of animals tended to seek more novel types of social-state configurations.

4. Finally, the authors examined circadian patterns in both adult and pup behavior. The claimed/showed: gerbils had a slight preference towards expressing more behaviors during the day cycle. Adult behaviors would ramp up during the daytime, with the exception of adult-adult proximity. Exploration and pup-time would ramp up during the daytime during all developmental periods, while this gradually developed in other behaviors, such as food hopper & waterspout

exploration, distance traveled, and pup-pup approaches. A circadian pattern in behavior becomes more visible with development in pups in metrics of exploration time, distance traveled, food hooper proximity, and pup-pup proximity time. Circadian patterns developed (or possibly became easier to detect) over development for metrics of food hopper proximity, pup-pup proximity time, exploration time, and distance traveled. The authors argued using data and models of data that development had three phases with transitions between P16 to P19, P20 to P27, P28 to P29. It is not clear if P29 is a cutoff or if this is simply where measurements end due to data collection.

5. Strengths of the manuscript include: -Detailed tracking of behaviors in individuals across early development using enlarged home cage -An interesting focus on the transition to independence -Analysis of complexity of social states among multiple animals across development -Full day allows capacity to analyze temporal profile of behavior to examine circadian and other patterns

6. Weaknesses

7. A. The lack of male subjects and small subject number overall makes sex differences section of the results too weak to be reliable. Individual differences/litter differences may also be driving these results.

    a. We agree with the reviewer and have removed sex-based results from our manuscript.

8. B. There is also no analysis of weight and weight gain and its relationship to behavior. Were heavier animals faster to develop? Did changes in feeding/hopper approach correlate with a growth spurt?

    a. We unfortunately did not maintain this information as we sought to not interact with or perturb the pups and adults over the period of our study.

    b. The major finding we see on growth spurts is that water-approaches spiked substantially over a period of 2-3 days (P24-P26) coinciding with the female drinking increasing less.

9. C. Sociality is central to the manuscript but there is no analysis of dominance behaviors between sibs and/or links between different types of events and interactions. Are they guarding the hopper or just eating? Do they avoid each other after a scuffle? Do they approach those that weigh less or more? Are approaches aggressive (making another stand down) or affiliative? How does dominance affect approach and changes in approach? Also, how are gerbils different or unique in their behavior compared to other rodents. Hamsters, rats, and mice? Voles? Do they have play to aggression transition like rats? Are they precocious ?

    a. We thank the reviewer for these comments. We have made several findings regarding pup interactions within the hopper region such as spending up to two times longer there than adults starting around P26, despite traversing less distance than adults across all stages of development. We also showed that pups approach the adult female about twice as much in late development than the adult male overall.

b. However, machine vision tools have not yet been developed to classify complex behaviors of multiple animals that are freely behaving as in our study (e.g. guarding vs. eating near the nest, or complex interactions such as fighting or playing. We did not notice any asymmetric interactions between pups to suggest dominance hierarchies were available from our behavior measurements. Overall, adding such analysis would require significantly more work, including substantially more machine vision tool development, and we view as beyond the scope of our study. As our study is unique we are releasing all of our video and animal tracking data to the community as part of our peer-review process as there is indeed a need for such behavioral datasets in the machine vision and deep learning development community.

c. With respect to comparing these behaviors and other rodents, there are no other studies similar to ours that track with second resolution the behavioral classes that we identified.

10. D. It is unclear if P29 is cutoff because this is where sampling ends or if this is a time point where aspects of behavior become stable into adulthood. This cannot be known without more sampling in older ages.

    a. P29 was the end of our study as pups need to be weaned shortly after (P30). Indeed, we agree that longer studies could provide more insight into how pup social and individual behavior dynamics converge back into adult-like behavior.

11. Further Questions

12. E. It would be interesting to see data from recording with the ultrasonic mics to see how vocalizations changed across development, varied with day/night cycle, and interactions between adult & pup vocalizations

    a. We have published vocalization work separately Peterson et al 2023 as it was a different project (https://elifesciences.org/reviewed-preprints/89892).

13. F. I didn't really see the need for the extra blocks in the home cage environment as they weren't listed as a possible influence for behavior exhibited by either the pups or adults (i.e., novel objection interaction and how that developed over time). Not sure if the blocks were just for environmental enrichment, if they expected to see novel object exploration and didn't observe it, or if it was observed and not mentioned in the results

    a. We thank the reviewer for their comments. Indeed, the blocks were meant for both environmental enrichment and behavior analysis. We had trained neural-networks to also track the blocks and spent a significant amount of time on this analysis. However, we ran into several challenges with overtraining of NNs for blocks and did not disclose this analysis as part of our manuscript.

14. G. For approach behaviors: Expressing everything as a % of adult seems to break down in its utility here. Also unclear if the instigator of the approach matters or not. Adult to pup or vice versa.

a. We were particularly interested in the relative behavior of pups to adults and chose this particular normalization metric.

15. H. I am not familiar with complex hull overlap and also wonder what we might learn from more standard regression or correlation tests.

a. We thank the reviewer for this suggestion. Indeed, we could have also computed correlations across behavior types. We have computed some of pairwise correlations plots (as provided to the Editor comments) and showed that identity shuffling lead to significant lower within-behavior correlation vs. the real data (see water vs. water and shuffled versions in the plots below). Correlations across behaviors, such as water vs. food proximity (green scatter plots below) did have lower correlations than within behavior (e.g. blue and red scatter plots below). However, the overall correlation across these types of behaviors was confounded because they were correlated with latent causes such as an increase drive to seeking independent sustenance which unfolds on similar time scales.

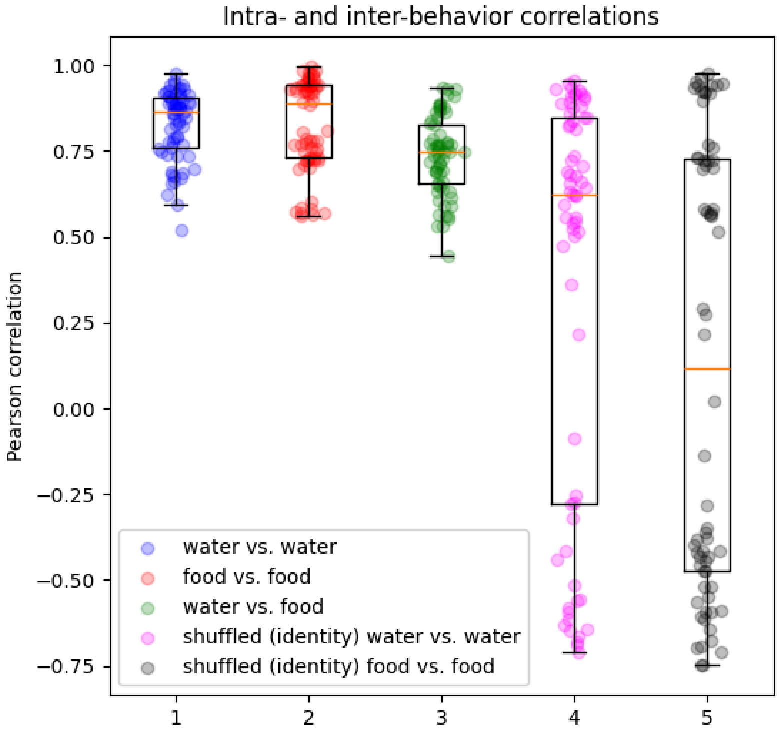

Supplementary Fig #5. Correlation distributions for all pair-wise individual pup developmental trajectories over 14 days of development for water and food.

b. Thus when comparing other behaviors with food and water we also generate highly correlated or uncorrelated behaviors (see figure below). This is essentially because correlations are a coarse measure of similarity that relies on the relative value of points in a time series to the mean. As many of our behaviors monotonically increased or decreased in similar ways, we did not view that correlation was a sufficiently nuanced method of analysis for our work. We are, of course, open to providing additional plots in our final manuscript explaining these results.

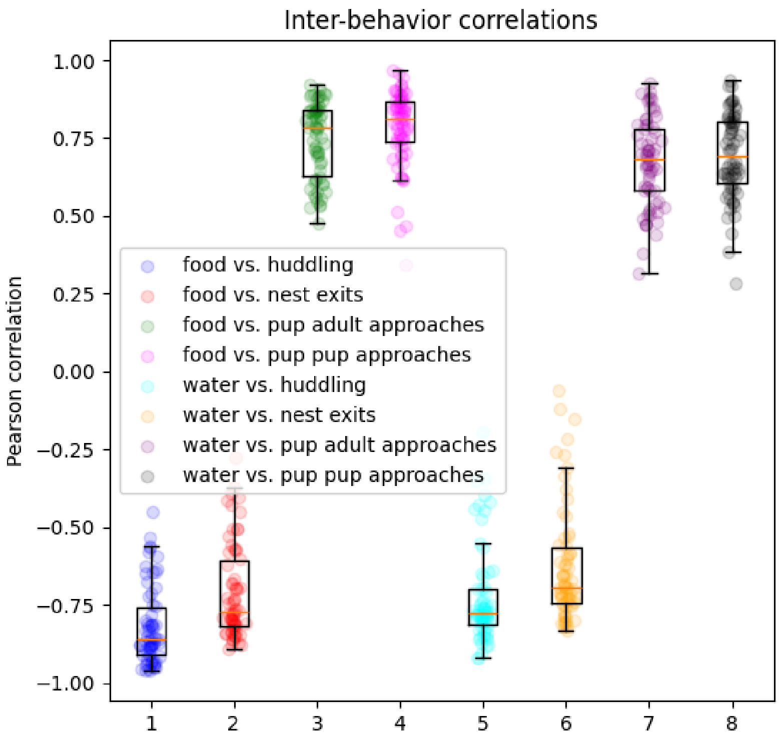

16. I. Statistics reporting was not always clear. For example in the sex differences sections, I wondered why the ttest results were not reported at the end of the sentences where the mean and stdev (or is it SEM?) was reported, but rather where difference is reported. Are these comparing 3 vs 9 mice or treating different days as independent samples to enhance power?

    a. We thank the reviewer for this note. We have now removed the sex difference sections from our work. For clarification, we sought to report all t-test and K-S tests results and it may have been an oversight on our end.

17. J. Some conclusions are not well supported. For example, it is unclear why ramping of behavior in light cycle would be considered by the authors to be related to genetic rather than learned (or experience dependent) causes.

    a. We agree with the reviewer and have removed this statement from our Discussion.

18. K. For reference in intro or discussion, can the authors comment on when are pubertal milestones beginning in gerbils? Also, do they have adrenarche and when?

    a. We thank the reviewer for their questions, this is an obvious issue we should have addressed. We have now added the following text to our discussion.

    Pubertal milestones in gerbils typically occur between 30-50 days of age. In females, vaginal opening usually happens around 30-40 days, followed by the onset of estrous cycles, while in males, preputial separation, marking reproductive maturity, occurs around 40-50 days. Adrenarche, which is the early maturation of the adrenal glands and

androgen production seen in humans, is not well-characterized in gerbils or most rodents. Rodents typically undergo a single, coordinated pubertal process without a distinct adrenarche phase and there is little evidence that gerbils experience adrenarche as in primates.

19. L. How do these behaviors relate to dispersal in the wild in gerbils? When do they disperse and which sex disperses farther in this species?

    a. We thanks the reviewer for this question as well, and have added the following text:

        In wild gerbils, dispersal from the nest typically coincides with the onset of sexual maturity, around the time of puberty. Juveniles often begin to disperse at around 6-8 weeks of age, shortly after reaching sexual maturity. Dispersal is driven by a combination of factors including competition for resources, territory, and mates. Males usually disperse farther than females in many gerbil species. This is likely due to the reproductive strategy of males seeking mates outside of their natal territory, while females tend to stay closer to home to establish their own breeding territories.

20. M. Page28 It is not clear to why is this food hopper behavior development considered a transition in non-circadian to circadian? Could other changing aspects of behavior -not related to circadian changes- generate these t1 and t2 transition points? What measure is this focused on

    a. We thank the reviewer for their comment. Indeed, it is possible that other developmental programs could drive the changes observed but were not captured in our methods. Our goal was to evaluate whether qualitative observation of three periods of development would be observable in the circadian rhythm analysis. We thus developed a greedy algorithm that sought to maximize the differences between three periods of development and reported t1 and t2 - which are the times that generate the largest "separation" between these 3 periods. However we agree that additional behavior developments could contribute to these transitions and have added the text to our section:
        i. We note that this analysis relies on heuristics and assumptions noted above and additional confounds may explain these transitions rather than an innate circadian rhythm development program.

21. N. Page 28 hopper typo (hoper)

    a. Corrected.

22. O. Page 29 "Inner-behavior" was this something else?

    a. Corrected to "inter-behavior"

23. P. "Stabilizing" is maybe not right word for some patterns. If a behavior stabilizes at 200% of adult, then it must come down later when not observed.

    a. Changed to "reached"

24. Q. Page 30 The authors state "We speculate that social grouping in pups may, in fact, be a platform for developing foraging and exploration skills." No references are given. I believe there may be others who have discussed this previously for rodents and certainly there are parallels in humans. For a rodent example, JB Calhoun in the Psychology and Ecology of the Norway Rat which is not referenced despite rich parallels to the current study. mentioned. Also the book Wildhood, may help lead to more peer reviewed work across the animal kingdom on this topic.

    a. We thank the reviewer for their comments and have added these references to our work. JB Calhoun has been - and continues to be - a great influence on our work as we seek to expand neuroscience and neuroethological paradigms beyond head fixed and stimulus driven paradigms.

25. R. "Therefore, one plausible interpretation is that the highly dynamic and specific periods of development that progress from a minimal state to near-adult levels between P16 to P29 are driven, in part, by maturation of HPC-MEC axis." Next motor M1 is discussed, but no other regions are mentioned. This speculation in the discussion feels far too high level and specific, given maturation of other systems and threat related behaviors at this time too. For other examples, see Klune, Chin, Denardo 2021 which focus on cortex but also BLA. BLA plus septum and PAG may be important for exploration and possible avoidance of threat. Also why not consider the NAc and motivational systems. Relatedly, hunger and weight and growth spurts (see comment above about weight) could also affect behavior. Not to mention pubertal changes which are not mentioned. Also of interest non rod or cone negative phototaxis in young rodents which may wane: See Johnson, Wu, Donovan, Copenhagen 2010 https://doi.org/10.1073/pnas.1008533107

    a. We thank the reviewer for their detailed comments. Indeed our focus was too specific, and we have largely adopted some of the reviewers suggestions by adding this text to our discussion:
    While the maturation of the HPC-MEC axis and motor cortex may contribute to behavioral changes during P16 to P29, it is essential to consider other regions involved in development and threat-related behaviors. The BLA (basolateral amygdala), septum, and PAG (periaqueductal gray) play crucial roles in exploration and avoidance of threats during this time, as suggested by (Klune et al. 2021). Additionally, regions involved in motivation, such as the nucleus accumbens (NAc), likely influence behavior, especially in relation to hunger, growth spurts, and pubertal changes, which may affect exploratory behaviors (Wahlstrom et al 2009). It’s also worth noting that non-rod or cone-mediated phototaxis in young rodents (Johnson et al. 2010), may wane during development, potentially influencing behavioral responses to environmental stimuli.

26. Overall, the references cited could be expanded.

    a. We have added references.

27. S. "Inter-sex" may not be the best term to use for comparing males and females in methods since it has alternative meaning

a. Agreed, we have changed it accordingly (though this section has been removed from the current study).

Response to Reviewer #3:

1. Investigating early-life social behaviors is crucial for understanding development and brain maturation in social mammals. This endeavor is challenging as it requires precise behavioral monitoring over extended periods. In this study, the authors analyzed continuous behavior recordings of gerbil families and found that pups exhibit complex social behaviors from an early age. Independent behaviors like foraging emerge later. This work is significant as it provides novel insights into the early social development of gerbils and its potential role in brain development, and it also demonstrates a methodological approach for analyzing early social behavior in social mammals. However, I have several comments that should be addressed before publication.

2. Major Comments: 1. Generalization to rodents: The authors generalize their discoveries to rodents, as indicated by the title "Developing rodents exhibit structured social behaviors prior to the emergence of autonomy." I recommend limiting the conclusions to gerbils. Other rodents, such as mice or rats, may exhibit different developmental trajectories. As rats, mice, and gerbils show differences in their family life and development, caution in generalizing the discoveries across species is warranted.

    a. We agree with the reviewer and have limited our results to gerbils including changing our our title to reflect this:
        i. **Title:** Developing gerbils exhibit structured social behaviors prior to the emergence of autonomy

3. 2. Interpretation of data: The authors state, "Viewing the autonomous, social, and sex-based behaviors together - our interpretation is that pups depend on social interactions with other pups from an early age and are able to form specific attachments and representations likely from the nest that they carry out into foraging exploration. This suggests that the maturation of critical sensory-motor systems, which occurs post P15 in most rodents (Tan et al., 2017), may be sustained and possibly driven by social behaviors. We speculate that social grouping in pups may, in fact, be a platform for developing foraging and exploration skills." This interpretation is not fully supported by the presented data. The data do not show causality, and other plausible hypotheses could explain the observations. For instance, social interactions may be necessary for the development of specific social skills but not foraging behavior, which could be determined by increased caloric need. The data do not show what would happen if social interactions were restricted, making this conclusion unjustified. This should be discussed.

    a. We thank the reviewer for their comments. We agree that there are alternative explanations and that our conclusion needs to be caveated. We did not intend for this initial study to be perturbative or test causal hypotheses that would require significant manipulations of natural behavior of animals.

    b. As we have removed the sex-based differences section, we have also removed this statement from our Discussion.

4. 3. Sex differences: The conclusions regarding sex differences should be moderated. Among the three families studied, there were only three male pups and nine female pups, resulting in a

very small and unbalanced sample. The data regarding social approaches are highly variable. For example, "male pup to male pup approaches occurred an average of 30.07 ± 20.64 times/day (cohort 2 data only), and female pups approaching female pups occurred 20.01 ± 16.66 times/day." The data for male pup-male pup interactions come from one cohort, involving interactions between two males. Therefore, the results of sex comparisons should be interpreted with caution.

   a. We agree with the reviewer and other reviewers and have removed the figure and sections on sex-differences.

5. 4. Cohort differences: Potential differences between different cohorts of animals should be shown. The varying composition of groups regarding pups' sex may have effects on the analyzed behaviors. This factor should be mentioned in the limitations section of the discussion.

   a. We thank the reviewer for their suggestion. In our experimental set up we did not observe substantial changes between cohorts. We have added a statement regarding this in our Discussion:
      i. While there were different numbers of female and male pups in each cohort, we did not find substantial differences between pup behaviors from the different cohorts.

6. 5. Influence of excluded time points: Some time points were excluded from the analysis due to cage cleaning (P24 or P18), and behaviors were interpolated from the days before and after these time points. Does this influence the analysis? This is important, as these days are identified as the middle of critical periods. It is possible that these days appear more similar to neighboring days due to estimation, potentially affecting the critical periods identified by the analysis. This should be discussed.

   a. We thank the reviewer for their comment. We have noted in the limitations that data replacement due to cage cleaning could have affected some of our metrics which relied on stable or rapidly increasing behaviors over days.

7. Minor Points: 1. Figure 3: The term "psycho-social driven behaviors" should be changed to "social behaviors."

   a. We have made this change.

8. Figure 2: The colors in the figure are difficult to distinguish and should be made more distinct.

   a. We will update the colors on acceptance of the manuscript.
