# Supplementary material for: Continuous monitoring and machine vision reveals that developing gerbils exhibit structured social behaviors prior to the emergence of autonomy
Source: PLoS Biol. 2025 Sep 8;23(9):e3003348. doi: 10.1371/journal.pbio.3003348 (PMC12416696; doi:10.1371/journal.pbio.3003348)
Supplement: Attachment — Submitted filename: Response_to_Reviewers_June_9_2025.pdf [file pbio.3003348.s009.pdf]

Dear Roli Roberts and reviewers

We thank the editor and the reviewers for their detailed comments on our manuscript. Below we provide detailed responses to the raised issues and explain the modifications we've made. We look forward to working with PLOS to complete the resubmission process.

Reviewer 1

1. Incorporation of Supplementary Figures: The rebuttal letter references supplementary figures that provide interesting insights. However, unless I missed something, I do not see references to supplementary material in the main manuscript. Why is this material not included in the paper, or at least some of it? If these figures strengthen the main findings, it would be beneficial to integrate them.
    a. We have now added a Supplementary Information submission with 6 figures. We have referenced the 6 figures in the text of the manuscript as Supporting Fig S1 and so on.

    b. Re: power analysis, we have now added another section to our Methods
        i. ***Cohort and animal group sizes power analysis.*** *To achieve a power of at least 80% with a significance value of 0.05 we estimated the required sample sizes for our work are 3 individuals in each group of pups and adults - whereas we had 12 and 6, respectively. Briefly, we estimated these values based on existing literature that shows P16 gerbil pups display little movement outside the nest, and a small variance of 10% relative to the adult variance of ~20%. Thus, for early development, 3 individuals in each group were sufficient, with 12 and 6 animals in each group providing power of ~90% or better for both 0.05 and 0.01 significance. Since we trained our neural networks to identify pups and adults across all days of our study (P16-29,) our cross-validation analysis applies to all age groups - not just early development - and we found strong evidence that the machine-vision tools generalize sufficiently to keep the same animal identity over the entire window of development. To summarize, our data and analyses support that groups of ~3 individuals each would provide sufficient power for this study.*

2. Long-Term Tracking and semi natural environment References: The manuscript would benefit from referencing additional studies that have tracked behaviors over extended periods in semi natural environments, even if it is not an analysis at a sub second scale. Relevant works include among other Shemesh et al. (2013), Freund et al. (2014), Torquet et al. (2018), De Chaumont et al. (2019), Forkosh (2019)…. Citing these studies does not diminish the novelty of the authors' recordings but rather strengthens the context of long-term behavioral tracking in rodents.
    a. We thank the reviewer for these, and have now added these citations and additional discussion to the final paragraph in which we cite these pioneering studies.

3. Definition of Solitary Behavior in Introduction: The term "solitary behavior" should be clearly defined, particularly since "agency" is discussed earlier. Here is my understanding: Solitary behavior refers to actions that an individual performs alone, without direct social interaction. But it is not non-social behaviour. For example, an individual may develop self-sufficiency or problem-solving skills independently while still being part of a social group.
    a. We thank the reviewer for thai clarification, we have now added this to Introduction:
        i. "*We operationalize social behaviors as those that involve approach or close physical proximity, and solitary behaviors as those performed without apparent coordination or cooperation with other family members*"

4. Ultrasonic Recording Mention in Methods: The sentence "Continuous video recordings were obtained from an overhead camera (24 fps), and audio recordings were obtained from four ultrasonic microphones (125 kHz sampling rate)") is redundant and should be removed as it a repetition of what has been said before. Furthermore, since ultrasonic recordings are not analyzed in the paper, all references to them should be removed from methods section, and image (Figure 1) ?.
    a. We have now linked the paper that makes use of these audio recordings, we feel it is not appropriate to remove the microphones from the image as it would be an incorrect representation of the experimental setup.

5. If percentages are used in the text (for example page 7: We found that pup distance traveled per day started at 8.72 ± 1.71 % of adult levels (or 8.01 ±1.53 meters/day) and increased continuously until P26 where it stabilized near adult levels at 85.90 ± 13.08 % then figures should also use percentages rather than values relative to adult averages—or vice versa.
    a. We have changed all values to reflect proportions (i.e. 0-1).

6. Clarification of Sample Size (n=12): When variances or standard deviations are estimated on 12 individuals, explicitly state n=12 in figures. Also, clarify whether the reference of 1 is obtained by averaging the two adults per cage.
    a. We have added this statement to the Methods:
        i. ***Computation of standard deviation.*** *Standard deviation reported in all results in brackets is for all pups, i.e. n=12. The values for each pup was obtained by averaging the behavior of the 2 adults within each cohort.*

7. Pairwise Social Exploration and Nest Time: Was pairwise social exploration analyzed in relation to time spent in the nest?
    a. We did not analyze social exploration against time spent in the nest. Though they are partially anticorrelated, the main finding was that pups socialize significantly more with each other than with other adults.

8. Were day and night phases accounted for?
    a. We did not split the analysis by day and night.

9. Additionally, were sleep periods near each other counted as social interactions? These points need clarification.
    a. We did not count sleeping periods in the analysis of social interactions.

10. Calculation of Time Spent in Groups: Provide details on how time spent in groups of adults was calculated. In the sentence: "We found that pups socialized in groups of three gerbils 6 to 7 times more than adults did in early development when they left the nest (i.e., as early as P16) and leveled off around 2 times as much by P29 (Fig 3h)." What is the reference for adult grouping? Since there were only two adults per cage, it could not have been groups of three adults—so what was the comparison based on? Clarify the baseline used for this analysis.
    a. We understand the issue and have added the clarification in the Methods as follows:
        i. ***Computation of group socialization time.*** *We computed the amount of time pups and adults were socializing as the time they spent within 5 cm of each other. This method was used for both pups and adults, for example, to compute how much time each age group spent near groups of 2 or 3 other animals (i.e. < 5cm from nearby gerbils). The relative time computation was computed similar to other steps, namely, by computing the socialization time for adults near other gerbils including other adults and pups and using this as a baseline.*

11. There is no reference to Figure 3j in the text.
    a. We have added a reference to Figure 3j.

12. The 3D PCA space used in Figure 2c is not sufficiently defined in the Methods section. If my understanding is correct, the analysis starts with an m × n matrix, where m (rows) corresponds to all pups (N=12) across four behaviors (4 × 12 rows), and n (columns) represents 14 days. The data used appears to be from Figure 2b (normalized to adult levels). If so, confirm in the Methods section.
    a. We have added this text to the Methods:
        i. *We computed the overlap using principal component analysis (PCA) as the intersectional volume of the first 3 principle components (using the python vedo package) or when this was not possible due to insufficient points to form a polyhedron - the first 2 principle components. The data input into PCA was [n_animals, n_time_points] = [12,15] where the values were normalized to adult levels.*

13. Also, does the repeated use of the same animal for four rows in the matrix introduce unexplicit dependencies in the PCA analysis?
    a. We have used the same animals for this analysis but different behaviours as this figure is aimed at explicitly evaluating the difference between the individual behaviour manifolds across all mice. The goal was to show that the developmental trajectories are qualitatively and quantitatively different. PCA can also be run on individual behaviours for the cohort - but this would not provide this information.

14. Clarification of "Distinct Trajectories" Statement: The sentence "This supports that the development of individual behaviors have distinct trajectories - rather than being explained by coming from pups in the same cohort". Thanks for adding this sentence but to fully support this

claim, an analysis of trends within each cage would be needed. If all pups in a given cage show similar trends, then cage environment might be influencing behavior. A simple way to show that would be to color members of the same cohort differently in figure 2c to confirm that points are not clustered by family?

a. We thank the reviewer for this comment and have now also added Supplementary Fig 6 based on the plot below. We have re-analyzed the results and provide a comparison plot below that does show some within cluster similarity between gerbil pup behaviours (note this plot uses different colours and viewing angle to maximize observation). This partial clustering result is indicated by the proximity of trajectories (i.e. individual points) for gerbil from similar cohorts (denoted by shape). However, our main conclusion that pups from the same - and different - cohorts have substantially distinct behavior trajectories (in this higher-dimensional space) is preserved. We have accordingly removed the term “significant” and added a qualifier to our conclusion as follows:

i. *In sum, we found that across four behavioral measures we could identify distinct developmental time courses that were different from each other, and from those displayed by the adult parents. We note that there was some within-cohort similarity between trajectories and further analysis could be carried out for this. Thus, food and water consumption developed on different timelines and had their own unique periods of rapid change, suggesting complex interactions between drives for nutritional needs and independent exploration of the environment.*

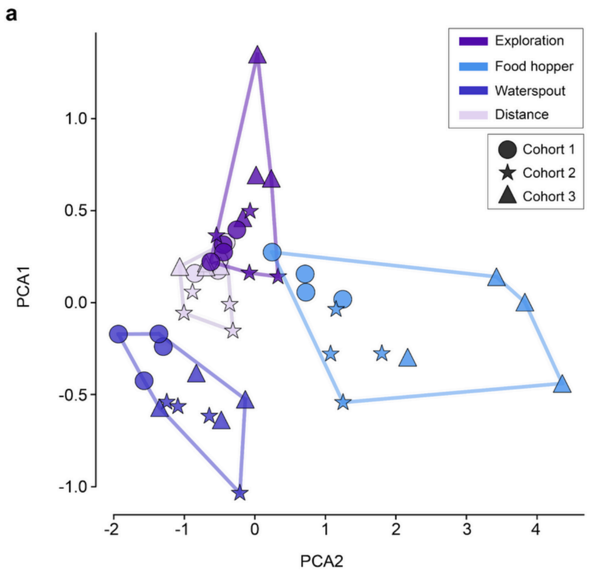

15. 12) The conclusion states: "Our findings reveal novel time courses for development in rodents, including dynamic periods of behavior acquisition, sex-based behavior differences, and the development of circadianity."

a. We removed "sex-based behavior differences".

Reviewer #2:

1. General comment: Overall, the statistics in results and figures could be more explicitly explained and reported. Currently, it is hard to fully follow how conclusions were supported and what comparisons were made and deemed to be significant.
    a. We identified all conclusion statements, and made certain to refer to a specific figure panel as the basis for the statement.

2. "We note that both distance traveled and environment exploration time underwent rapid development for most of the days between P17 and P25." Did the authors want to back this up by reporting the KS tests used?
    a. We used a 2-sample KS test to evaluate the differences, we provide a section in the Methods:
        i. ***Rapid development and stabilization periods.*** *We defined a rapid development period as Pdays that had distributions that were statistically significant than the previous 2 Pdays. We thus pooled behaviors in groups of 2 days and compared them following groups. We used two days as a more conservative way to account for higher variability - than applying smoothing or other filtering techniques on the raw data. We used a 2 sample Kolmogorov-Smirnov test as a more conservative measure of significance. For clarity, at each Pday, we pooled data from individuals (e.g. 12 pups or 6 adults) and computed the statistics across these values. We used the python package statsmodels method sm.stats.multipletests for the Benjamini-Hochberg multiple hypothesis testing adjustment and reported pvalues < 0.05 as statistically significant.*

2. In the figures there are delta(triangle) symbols, but they are not explicitly linked to a significant KS test by this comment "with indication of rapid development days" in Fig 2. The symbols are in Fig. 3 legend too but are not defined there.
    a. We thank the reviewer for this oversight, we have now added a note to the figures that an explanation of rapid development days is provided in the Methods, as shown above.

3. "Similarly, pups were followed out of the nest by parents significantly more than parents followed other parents on P16 at 151 ± 0.17 % (and overall followed 76.09 ± 8.57 % of the time), and this nest following behavior stabilized also around P24 at 107 ± 12 % (or 53.53 ± 5.88 %) (Fig 3b)" Statistical tests done here unclear.
    a. We removed the word “significant” from this statement as it was a qualitative statement, thank you for the comment.

4. Authors in results: "This suggests that increased group sizes could be supportive of environmental exploration and that single animals or smaller groups tend to use more stereotyped or repeated trajectory patterns." Does this correct for chance?
    a. We have not carried out extensive controls and shuffled conditions on this result. We added a qualifier to our findings as follows:
        i. *However, additional computational analysis including more in-depth shuffling and controls may further extend this result into novel directions*

5. Circadian section is also lacking statistics for group comparisons.
    a. Are there any statistics?
        i. We have largely reported qualitative findings in the circadian behaviour analysis.

    b. There seemed to be differences in peaks in night for one period for adolescents. Were comparisons made so age period was paired between adult and adolescent groups (since activity one might affect the other)?
        i. Our general conclusion from Figure 5 is that the circadian pattern displayed by older juveniles (P21-25) and adults is quite similar across a broad range of measures. While young juveniles (P16-20) differed on some measures (e.g., time in foodhopper region), this was inevitably linked to a failure to display the behavior, rather than differences in circadian pattern.

    c. Unclear to be How is circadian behavior (or non circadian) behavior defined and tested?
        i. We have additionally clarified our method as below. Briefly, 3 age groups were identified based on observations in fig 2, and we then performed a greedy search for optimal separation of circadian behaviour. We added the following to the manuscript
            1. In the main text:
                a. *We used a computational approach to agnostically identify the age at which behaviors transitioned from non-circadian to circadian patterns.*
            2. In the Methods:
                a. ***Automatic detection of circadian rhythms development**. We developed a computational method to evaluate whether pups had more discrete transitions from non-circadian to circadian rhythms-driven behaviors. The method was applied to food hopper region proximity data - and an assumption of three developmental periods based on qualitative observations in our study defined as: an initial period D1, a middle period D2 and a final period D3. Based on our qualitative observations we initialized these periods as: P16-P20, P21-P25 and P26-P29. We next sought to determine whether the boundaries on these periods, i.e. t1 and t2 corresponding to P20 and P25, respectively, could be quantitatively defined via an optimization function. The optimization function sought to maximize the difference between the individual animal circadian trajectories in each of these periods. Our algorithm identified t1=19 and t2=27 as the dates that create the maximum differences between pup presence in the food hopper region, i.e. D1: P16-P19, D2: P20-P27, and D3: P28-P29. (Fig 5h). We noted that different behaviors or priors, e.g. a single transition - may provide slightly different developmental windows.*

6. Interpretation of data varies by section. Authors in Results note 100% more time in food hopper region in late P20s pups. They interpret this in results with "This suggests that in addition to learning the nutritional value of the food hopper region, pups may also value this space as a socialization space significantly more than adults (see more below)." (Alternatively, do they just need to eat more? Was this due to 2nd period nighttime eating seen in circadian figures?)
    a. We agree with this statement, it is quite possible as we did not observe evidence that the gerbil families hoard food. We have added this statement to our findings:
        i. *However, it is also possible that young gerbils also spent more time at the food hopper as they may have consumed more calories during development.*

    b. Later in the discussion they say "These findings provide independent confirmation that the pups' increase of food hopper and waterspout proximity were driven, in part, by consumption behaviors and a decreased dependence on the adult female."
        i. We have removed this statement from the manuscript.

7. In the discussion, there is almost no development of the social ideas emphasized earlier in the manuscript and in the abstract (claim "Our work supports a model in which early and sustained social interactions may be supportive of solitary exploration for physiological needs"). If the authors want to invest more in this social support idea, I think they might add in studies that posit social influences on behavior in other species? I think this is in literature coming from primates (great apes), dolphins, maybe elephants who also show youth groupings weaning to juvenile phase. If gerbils have complex social structure this could be interesting link. doi.org/10.1016/j.anbehav.2017.04.011
    a. We thank the reviewer for this suggestion, we have added a paragraph to the Discussion section to address this topic as follows:
        i. ***Early social interactions support the transition to independence.*** *Juvenile and adolescent animals often display a rich array of social interactions with their peers, and this may serve as the substrate for the emergence of adult skills, especially in advanced mammals (Pereira and Fairbanks, 2002; Vanderschuren et al., 2016; Krzyszczyk et al., 2017). However, the long-term effects of experimentally manipulating early social experience have largely been carried out in rodents. For example, depriving pre-adolescent rats of social play for two weeks leads to decreased social activity in adulthood (Hol et al., 1999; van den Berg et al., 1999). Furthermore, juvenile play during a developmental critical period can induce a preference for the environment in which the social interaction occurred (Calcagnetti and Schechter, 1992; Panksepp and Lahvis, 2007; Nardou et al., 2019), and this effect is diminished when pups are treated with a drug that interferes with social play (Trezza et al., 2009). Our descriptive data is broadly consistent with a constructive role of early social experience. When gerbil pups begin to leave the nest, they preferentially retain sibling interactions (Figure 3d, h,and i), which then leads to a dramatic increase in open exploration time (Figure 2b).*

8. Claim in discussion: "This supports that the development of individual behaviors have distinct trajectories - rather than being explained by coming from pups in the same cohort."

a. This is an interesting point that could perhaps be more thoroughly supported in explicit terms. For example, we do not get to see any plots of the three different cohorts next to each other and the order of their relative developmental sequences.

    i. We thank the reviewer for their suggestion. Inter-cohort analysis was not the focus of our study. We have changed this wording to a more general conclusion as we do observe slight within-cohort similarities for behavior trajectories - though our main conclusion was that the behaviours largely occupied different trajectory spaces is not affected:

        a. *In sum, we found that across four behavioral measures we could identify distinct developmental time courses that were different from each other, and from those displayed by the adult parents. We note that there was some within-cohort similarity between trajectories and further analysis could be carried out for this. Thus, food and water consumption developed on different timelines and had their own unique periods of rapid change, suggesting complex interactions between drives for nutritional needs and independent exploration of the environment.*

b. Also were pup weights (as a proxy for developmental advancement) comparable between cohorts at the start of the experiment? Does this correspond to temporal rank in terms of the unfolding of events?

    i. We did not weigh the animals during the 2 week recording period because we sought a paradigm that restricted human interaction to the extent possible.

c. Relevant new study that might be cited Power et al., 2025 Animal Behavior (in press) https://doi.org/10.1016/j.anbehav.2025.123084

    i. We thank the reviewer for highlighting the Power et al. (2025) study, which offers timely and relevant insights. We have now added this additional paragraph.

        1. *Findings on cooperative winter nesting in voles appear to support some of our findings in developing gerbils. Scheibler et al. (2004) describe Mongolian gerbil burrows as complex, communal structures with large feeding chambers, especially elaborate during harsh winters—reflecting their non-hibernating, social lifestyle. Field studies further demonstrate substantial variability in burrow group size, ranging from 2 to 28 individuals (Leont'ev, 1954, 1962; Ågren et al., 1989; Liu et al., 2009), and emphasize that the dominant social structure comprises extended families, typically one adult male, one or two adult females, and their offspring (Gromov & Popov, 1979; Gromov, 1981, 1992, 2000, 2011). These ecological observations are consistent with our laboratory findings of structured, cooperative behavior in developing gerbil pups.*

Minor:

1. Typo: Worman=workman 2013
    a. We corrected this.

2. Between-sex statistical differences tests still in methods
    a. We deleted this comment.

Reviewer #3

1. I have just one minor comment: in the Discussion section (p. 20), there is a reference to sex-based behavioral differences, but since this part of the results has been removed, the statement no longer fits.
    a. We have deleted this.
