# Supplementary material for: Continuous monitoring and machine vision reveals that developing gerbils exhibit structured social behaviors prior to the emergence of autonomy
Source: PLoS Biol. 2025 Sep 8;23(9):e3003348. doi: 10.1371/journal.pbio.3003348 (PMC12416696; doi:10.1371/journal.pbio.3003348)
Supplement: Attachment — Submitted filename: Response_to_reviewers_revision_July_28_2025.pdf [file pbio.3003348.s010.pdf]

Dear Roli Roberts, PLOS Biology Editors, and Academic Editor,

We thank you for your detailed comments on our revised manuscript. Below we provide detailed responses to the issues raised in your email, and explain the modifications we've made. We look forward to working with PLOS to complete the resubmission process.

a) Please modify the Title slightly (in both the manuscript file and in the EM metadata) to include the methodological approach. We suggest: "Continuous monitoring and machine vision reveals that developing gerbils exhibit structured social behaviors prior to the emergence of autonomy"

We have accepted the Editor's suggested title.

Continuous monitoring and machine vision reveals that developing gerbils exhibit structured social behaviors prior to the emergence of autonomy

b) The Academic Editor has kindly gone through your manuscript, and has made a large number of detailed recommendations (see the foot of this email). S/he said "I do think there's a fair bit of work needed to tidy this into shape from a paper-craft perspective (lots of methods and results and discussion and interpretation all mixed up, I don't mind a little contextualisation in longer papers but this does come across as a bit of an inconsistent hodgepodge at the moment, and there is direct repetition with sections that remain in the Methods). There are a few sections with a clearer polish in the discussion - but there is a fair bit of work to be done to showcase their interesting findings in a light that the reader can easily follow along with." and "at times there was so much ambiguity in what they were saying that it was really challenging for readability." We strongly recommend that you address all of the Academic Editor's requests and get a colleague who is a native
English speaker to give the manuscript a final go-through.

We have implemented each of the Academic Editor's suggested basic writing edits (a line-by-line response to the writing edits are presented at the end of this letter).

Our responses the the Academic Editor's queries/non-linguistic points were addressed, as follows:

Line 107 -> 0.91 true positive rate for pups, suggests a 10% error rate in identification in their frames, given how few individuals there are (and how many frames) this is a little concerning. I'm not sure if they assessed whether this was randomly distributed, or if there were particular errors (e.g., two individuals were swapped systematically more frequently, which would be more problematic).

We add the notation that chance identification is 0.17. This suggests that we are able to identify animals substantially above chance. We did not find any evidence of specific identity swaps.

Typesetting check -> they've sometimes added bolding, I'm not sure if this is for emphasis, but I'd suggest that these are all removed (except titles etc.).

All extraneous bold text has been eliminated

Line 160: there is an inference here about the social importance of the space, relative to adult use (as well as the nutritional value) but I'm not sure on what basis that is drawn? Is it only that they're there twice as often as adults? Couldn't that be to do with eating patterns (feeding more slowly? Etc.)

We have removed this interpretation concerning the relative social value of the food hopper space.

In almost all cases they give qualitative descriptions of patterns in the data, but I'm not sure why they don't test some of these in a quantitative manner? e.g., all the values on page 10 of pup-pup vs pup-adult interaction time could be tested with very straight forward measures that would let them say with greater confidence whether there was anything there beyond potential patterns.. I hate to ask for additional tests at this point, but given regular use of terms like 'significantly more' they seem interested in making these claims. If they'd rather not, then I think more work is needed to de-emphasise the behavioural findings and interpretation.

We have chosen to de-emphasize or eliminate qualitative statements and many of the interpretations within the Results section.

Line 377: you used social and solitary in the intro, do you mean the same thing here with social and autonomous?Line 406: I would avoid 'advanced' mammals, there aren't really more/less advanced species

Yes, "solitary" and "autonomy" are used interchangeably. Thus, we have slightly modified the Introductory sentence in which operationalize social and solitary behaviours to include the term autonomous:

> *"We operationalize social behaviors as those that involve approach or close physical proximity, and solitary behaviors as those performed <u>autonomously,</u> without apparent coordination or cooperation with other family members."*

We have removed the sentence modifier, "especially in advanced mammals"

Line 407: Harlow conducted a long and infamous series of experiments on early social deprivation in primates

The sentence introducing rodent manipulations has been modified to reference two of Harlow's social deprivation studies.

Line 461-465: a possible contribution to the variation with field studies would be the lack of predators in captive contexts..

We have added a sentence that notes two significant differences between field research studies and our paradigm: the absence of risk, such predation, and presence of ad libidum food and water.

Line 481: could the absence of underground areas also impact circadian rhythm development?

Yes. We have added a sentence citing an lab study in which animals were reared with access to tunnels which influence the emergence of normal escape behavior.

Line 591: dimensionality is indeed a curse, but perhaps there is a more formal way to refer to this..

We have modified the sentence.

b) Please supply the protocol approval number from the NYU Animal Welfare Committee.

Protocol 2020-1112

c) Please supply a link to your funding agency in the Financial Disclosure Statement.

Funding agency link: https://www.nih.gov/, https://ethics.od.nih.gov/fd

d) Please address my Data Policy requests below; specifically, we need you to supply the numerical values underlying Figs 1D, 2BCD, 3BDFHI, 4FHJ, 5BCDEFGH, S1, S2AB, S3AB S4, S5, S6, either as a supplementary data file or as a permanent DOI'd deposition (e.g. as part of your Dryad depositions).

We have now submitted our raw data and tracks, as well as metadata for the request figures to Dryad. Laura Bowman, Senior Data Curator II is helping us finalize this as we are requiring close to 1TB of space for raw videos. The reviewer link below should be accessible now.

e) I note that although you already have two associated Dryad depositions, these are currently not accessible. Please provide a reviewer link so that we can check your compliance with our data policy.

The reviewer link is here

- https://datadryad.org/share/SAPvQOg6iVob2D9WrQYpIK27xyfdLIHdB8zAVNR54Hc

f) Please cite the location of the data clearly in all relevant main and supplementary Figure legends, e.g. "The data underlying this Figure can be found in S1 Data" or "The data underlying this Figure can be found in https://datadryad.org/XXXXX"

We have added a description that the underlying datasets can be found at the permanent Dryad link which is: https://doi.org/10.5061/dryad.t1g1jwt7w

g) Please make any custom code available, either as a supplementary file or as part of your data deposition. I note that you mention "custom postprocessing code available at https://github.com/catubc/gerbil" - because Github depositions can be readily changed or deleted, please make a permanent DOI'd copy (e.g. in Zenodo) and provide this URL.

We have uploaded our code base here: https://zenodo.org/records/16284382

Journal Requirements:

- We have noticed that you have a list of Supporting Information legends in your manuscript. However, there are no corresponding files uploaded to the submission. Please upload them as separate files with the item type 'Supporting Information'.

We submitted a Supplementary Information document containing both the Supplemental figures and legends that we refer to within the MS text. We have left the individual Supplementary figures in the submission site, and these can be deleted by the editors if our Supplementary Information document is sufficient.

Detailed response to Academic Editor's basis writing edits are interleaved below:
Line 41: social behavior -> social behavioral
accepted
Line 42: than anticipated -> than typically considered
accepted
Line 45: numerous survival and thriving goals -> goals allowing individuals to survive and thrive
accepted
Line 49: largely rare -> rare
accepted
Line 50: these two sentences don't seem to follow from each other, clarification needed?
We removed the second sentence that referred to interspecies comparisons.
Line 54: family social bonds -> familial social bonds
accepted
Line 57: motor (REF) and -> motor (REF), and
accepted
Line 58: emerge -> emergence; an attraction -> the attraction
accepted
Line 63: what is 'strong' social structure'? -> perhaps strong social relationships
Changed to "strong family social relationships"
Line 64: We operationalise.. -> Here, we operationalise
accepted
Line 65: cooperation with other family members -> cooperation with other individuals
accepted
Line 66: with a pair-bonded -> with pair-bonded
accepted
Line 70/71: (P) 16, and while -> (P) 16 and, while
accepted
Line 76: leave -> left
accepted
Line 78: behavior classifiers (REF) and -> behavior classifiers (REF), and
accepted
Line 80: preceding -> precede
accepted

Line 81: if you say more, more than what? (More …….. than is typically recognised)?
We did not understand this comment
Line 109: which are -> that were
accepted
Line 123: This -> These tracking results
accepted
Line 130: independently to -> independently of
accepted
Line 130: is it only the parents that are adults? If so cut adult, if not cut parent.
accepted
Line 138: relative to the adult -> relative to the adult mean.
accepted
Line 140: when it was -> in which it was; but also ‘it’ is ambiguous here, what does ‘it’ refer to?
changed to: “when that day was”
General note -> most of your uses of ‘computed’ can/should be edited to ‘calculated’
We substituted calculated for computed throughout the MS
Line 145: this sentence/method is unclear, I’m not sure what ‘this’ refers to, perhaps ‘of the variance’? Some clarification needed.
clarified
146: was more interpretable -> was more easily interpretable
accepted
147: distances which -> distances, which
accepted
Line 149: P26 where -> P26, where
accepted
Line 153: These open environment exploration findings -> these findings on open environment exploration
accepted
Line 157: to P21 at 0.45 ….. -> to 0.45 …… at P21
accepted
Line 158: between P22 until P26 stabilizing from P27 - P 29 -> between P22 and P26, and stabilizing from P27 to P29
accepted
Line 159: This -> This pattern (general note, unspecified this/these/it can be challenging for the reader to interpret)
We specify what the pronoun refers to
Line 161: do you mean that they spent more time eating while at the hopper? (This phrase makes it sound like they spend more time now, because earlier in development they consumed more calories -> clarification needed).
We have rewritten the sentence to clarify our interpretation
Line 164: with pups spending -> in which pups spent
accepted
Line 164: adult averages -> adult average

accepted

Line 166: access -> exploration (ideal to keep consistency in terms, unless you separately measured access here?)

accepted

Line 167: spend -> spent

accepted

Line 168: relative to the adults -> of mean adult time

accepted

Line 175: This what?

Sentence removed

Line 179: should decrease be decreased or decreases? (Unclear)

changed to decreased

Line 181: but does not affect -> but this behaviour clustering does not appear to affect

accepted

Line 183: measures we -> measures, we

accepted

Line 183: distinct developmental -> distinct gerbil pup developmental

accepted

Line 185: cut adult

accepted

Line 186: out for this -> out to investigate the effects of this

accepted

Line 186: Thus, food and water -> Food and water

accepted

Line 201: 0.02 (or .. -> 0.02 of the adult mean (or

accepted

Line 203: more than -> for more time than

accepted

Line 203: 0.02 -> 0.02 of the adult mean.

accepted

Line 203: stabilized also -> also stabilized

accepted

Line 208: please remove the word significantly (you don't seem to have tested this?)

accepted

Line 210 -> in several places in these results please specify where the value is 'of the adult mean' or 'of adult-pup mean' etc.

accepted

Line 214: please remove the word significantly (you don't seem to have tested this?)

accepted

Line 216: over all days -> across the full observation period

accepted

Line 217: show that while -> suggest that, while

accepted

Line 218: please remove the word significantly (you don’t seem to have tested this?)
accepted
Line 220: approaches with -> approaches, with
accepted
Line 221: this is closer to ~2 fold than to ~3 fold.. (the hours values are closer to ~3 fold)
accepted
Line 223-229: again please add ‘of adult means/average values’ etc. throughout for clarity.
accepted, and added throughout the Results section
Line 230 -> again as before, most uses of computed can be changed to calculated
We substituted calculated for computed throughout the MS
Line 231: 6 to 7 fold more -> 6 to 7 times more frequently
accepted
Line 232: off around 2 -> off at around 2
accepted
Line 234: This confirms -> This pattern suggests
accepted
Line 235: rather than being -> rather than partner preference being
accepted
Line 236: most behaviours, we find expected high -> behaviors, as expected, we find high
accepted
NB - please check for US/UK spelling consistency (e.g., behaviour vs behavior)
All changed to US spelling
Line 237: shape of the behavior -> angle of the behavioral trajectories
accepted
Line 241, 242: e.g. -> e.g.,
accepted
Line 243: substantially only at -> only increasing substantially at
accepted
Line 244: I’m not sure what integrity is used to mean here?
We removed this portion of the sentence
Line 262: This enabled -> This procedure enabled
accepted
Line 266: the social -> social
accepted
Line 269: suggest changing all use of animals in brackets to n=2, n=4, n=6
accepted
Line 270: sizes which represent -> sizes that represent
accepted
Line 272: please remove significantly (substantially?)
In general, please check over and remove all uses of the term significantly where there is no accompanying formal testing of the data.
accepted
Line 275: This suggests -> These differences suggest

accepted
Line 278: off around -> off at around
accepted
Please check all uses of computed -> computed implies a sophisticated calculation beyond the use of normal statistical methods (for which the term calculated can be used).
We substituted calculated for computed throughout the MS
Line 288: groups to be -> groups appears to be
accepted
Line 289: development or slightly decreasing suggesting -> development, or slightly decreasing, suggesting
accepted
Line 290: but rather were -> but were rather
accepted
Line 291: configuration -> configurations
accepted
Line 295: groups but not -> groups, but not
accepted
Line 295: We lastly computed -> Lastly, we calculated
accepted
Line 297: probability of a node to have -> likelihood that a node has
accepted
Line 302: configuration -> configurations
accepted
Line 305: 1.29 - suggesting -> 1.29; suggesting
accepted
Line 307: for exploration -> towards exploration
accepted
Line 312: This suggests -> These findings suggest
accepted
Line 315: ‘may further extend this result into novel directions’ -> I think this is better phrased as ‘would provide a more robust test of this result.’
accepted
Line 321: values were -> values are
accepted
Line 326: This suggests -> These differences suggest
accepted
Line 329: night cycle and similarly with -> cycle, and similarly for
accepted
Line 339: I’m not sure what ramping refers to in this section here, and below (perhaps increases? Some uses specify ramping up, please clarify).
We substituted “pattern” for ramping
Line 352: Fig 5f) and -> Fig 5f), and
accepted

Line 353: et al 1982). -> et al 1982.)
accepted
Line 354: circadian behaviors? perhaps circadian patterns (Also unsure why circadian is italicised in multiple cases).
accepted
Line 358: being mostly absent from -> being largely absent in
accepted
Line 360: double space typo
accepted
Line 369: was the most striking developmentally -> provided the most obvious differences in development
accepted
Line 371: (e.g. assumes 3 -> (e.g., it assumes three
accepted
Line 385: are more similar when compared with -> were more similar to each other, than when compared with
accepted
Line 386: This suggests that many classes -> This pattern suggests that these classes
accepted
Line 387: perhaps -> random and/or individual processes? (Given that you didn't find strong inter-individual differences)
accepted
Line 389: P26, when pups -> P26, at which time pups
accepted
Line 420: helpful to specify, 'all social and foraging behaviours commence at near zero' (it's a little odd to say that all behaviors start at near zero)
accepted
Line 434: -> For example, as compared to adults, gerbil auditory
accepted
Line 452: It's -> It is
accepted
Line 456: circadian rhythm effects -> effects of circadian rhythm
Could not find phrase "circadian rhythm effects"
Line 461: perhaps ramping up? (Again, ramping on its own is unclear out of context).
Note: please check all uses of 'ramping' in results and discussion, in general there is a need to specify when this term is used (ramping up, ramping down, on ramping etc. etc.).
Removed "ramping" from text
Line 474: compared to -> as compared to
accepted
Line 481: than the gerbil -> than gerbils'
accepted
Line 484: playing, sexual interactions which -> playing, and sexual interactions, which
accepted

Line 490: behaviors than other -> behaviors as compared to other
accepted
Line 505: orders of magnitudes greater?
Our description of the studies cited is accurate
Line 510: avoid undefined acronym in final sentence
accepted
Line 583: or when -> or - when
accepted
Line 584: into the PCA
accepted
Line 678: do you mean the comparison values for each pup or the values for adult averages? This sentence isn't clear.
We did not understand this query.
